# Multi-rate sampled-data control for T-S fuzzy systems with mismatched fuzzy basis functions

**DOI:** 10.1371/journal.pone.0355047

**Published:** 2026-08-07

**Authors:** Khanh Hieu Nguyen

**Affiliations:** Faculty of Electrical and Electronic Engineering, PHENIKAA University, Hanoi, Vietnam; Air University, PAKISTAN

## Abstract

This paper investigates a multi-rate sampled-data (MRSD) stabilization criterion for T-S fuzzy systems with the consideration of mismatched fuzzy basis functions (FBFs). To fully exploit the system information under a MRSD mechanism, we incorporate an improved looped-functional and a discontinuous function into the Lyapunov–Krasovskii functional (LKF). This construction explicitly leverages the most recent measurements and the sampling-induced delay associated with each system state. Furthermore, to address a set of conditions expressed as parameterized linear matrix inequalities (PLMIs), this paper develops a refined relaxation technique for mismatched FBFs that explicitly exploit their inherent properties. Finally, two examples are provided to demonstrate the effectiveness and practicality of the proposed method.

## 1 Introduction

In recent years, research activity on sampled-data control systems (SDCSs) has grown markedly, driven by the ubiquity of digital hardware and the need for high-precision, resource-efficient control in contemporary applications (refer to [1–3]). In this light, a substantial body of work seeks to enlarge the maximum allowable sampling interval (MASI) while preserving the stability of closed-loop system. Three principal modeling paradigms have emerged: the discrete-time approach [4,5], the input-delay approach [6–8], and the impulsive approach [9,10]. Within the input-delay framework, Seuret et al. [11] introduced a looped-functional, which avoids strict positive definiteness and yields less conservative stability conditions. Building on this idea, Zeng et al. [12] introduced a two-sided looped-functional to carry information across consecutive sampling instants. Subsequently, Lee and Park [13] proposed an advanced technique that incorporates a free-matrix-based integral inequality together with a time-dependent discontinuous term into the LKF. Thereafter, Lee et al. [14] derived a sufficient stability condition for SDCSs using zero equalities obtained by integrating the state equation over each sampling interval. In real systems, various state components are measured by different sensors with their own sampling rates, which naturally motivates the consideration of multi-rate sampling in SDCSs. In such settings, choosing the fastest rate oversamples slow channels and squanders computational and communication resources, whereas choosing the slowest rate degrades estimation fidelity and closed-loop performance. Therefore, MRSD with periodic sampling patterns has been investigated in [15–18]. However, because of multitasking in embedded platforms and time-varying delays in networked systems, strict periodicity is often difficult to maintain. To accommodate aperiodicity and asynchrony, Moarref et al. [19] established exponential stability and stabilization conditions for MRSD with aperiodic sampling in a multiloop controller for a quadrotor unmanned aerial vehicle (UAV). After that, Park et al. [20] developed stability and stabilization criteria for MRSD control systems via the looped-functional method. Recently, Yang et al. [21] proposed a fault-tolerant MRSD control framework for a quadrotor UAV. It should be noted that, in multi-rate sampled-data systems, three key factors must be simultaneously considered: (i) the specific sampling pattern of each state, (ii) the maximum allowable sampling interval for each state, and (iii) the sampling-induced delay from the last sampling instant to the current time for each state. However, the existing works [20] and [21] mainly consider only the first two factors, while the useful information associated with the sampling-induced delay has not been fully exploited within the LKF framework. In addition, although the results in [20] and [21] significantly enlarge the MASI compared with [19], their methods incur high computational complexity.

Meanwhile, the T-S fuzzy model is one of the most powerful methods for approximating nonlinear dynamics by blending a set of local linear models through normalized fuzzy IF-THEN rules (see [22–25]). The resulting overall system is is expressed as a convex combination of rule-wise linear subsystems driven by premise variables (e.g., states or measurable signals). This representation lets a wide class of nonlinear systems be handled within the LMI framework while retaining nonlinearity through the fuzzy basis functions. The controller developments for T-S fuzzy systems are supported by two complementary synthesis paradigms: (i) parallel distributed compensation (PDC), which assigns rule-wise gains blended by the same basis functions [26], and (ii) non-PDC, which allows premise-dependent but nonparallel gains [27]. To make these designs numerically tractable, Tuan et al. [28] introduced relaxation procedures that convert PLMIs into standard LMIs via variable elimination and new convex dilations. Recent studies have begun to treat MRSD control within the T-S fuzzy paradigm, tackling nonperiodic sensing and co-design of sampling bounds with stabilizing controllers. For instance, Huang et al. [29] developed an ${ℒ}_{2}-{ℒ}_{\infty }$ filtering method that uses a T-S fuzzy model and lifting to convert nonlinear MRSD dynamics into an equivalent single-rate fuzzy system. Then, Xu et al. [30] proposed a nonperiodic MRSD PDC framework for singularly perturbed nonlinear systems with slow and fast sampling intervals. However, the slow and fast partition used in [30] cannot capture the diverse sampling needs of large-scale systems with heterogeneous sensor. Recently, Ma et al. [31] investigated fuzzy dynamic output-feedback control for networked MRSD systems by developing refined integral inequalities and delay-dependent stability conditions. Moreover, Chen et al. [32] proposed an integral quadratic constraint-based dynamic output-feedback algorithm to address multichannel sampling and delay effects in networked control systems. In addition, Anbalagan et al. [33] developed a multi-rate sampled-data fuzzy consensus scheme for nonlinear multi-agent systems with time-varying delays and switching topologies. Despite these advances, most existing MRSD fuzzy control results focus on specific system structures or assume matched FBFs between the plant and the controller. In practice, however, multi-rate sampling mechanisms introduce asynchronous state updates, which may significantly enlarge the mismatch between the plant FBFs and the controller FBFs constructed from sampled data. Consequently, the mismatch problem becomes more severe than in the single-rate case. Nevertheless, the interaction between mismatched FBFs and multi-rate sampling has not yet been sufficiently explored in the literature.

Motivated by the above discussion, we develop an MRSD controller for T–S fuzzy systems that exploits richer information from each sampled state and explicitly accounts for mismatched FBFs. In summary, the main contributions of this work are summarized as follows:

- In contrast to [19–21], this paper constructs an improved looped-functional with additional delay-dependent terms and a discontinuous functional within the LKF framework. The proposed construction explicitly exploits the most recent sampled measurements and the sampling-induced delays of each system state in multi-rate sampled-data systems. Moreover, three zero-equality constraints are employed to introduce slack variables into both the stability analysis and the stabilization synthesis.
- Unlike existing looped-functional approaches developed for single-rate sampling, the proposed LKF is constructed directly for the multi-rate sampled-state structure. By selectively incorporating only the relevant state components associated with each sampling channel, the proposed method avoids the redundant decision variables that arise in single-rate-based extensions, thereby achieving improved stability performance with significantly lower computational complexity than existing approaches.
- In multi-rate sampled-data T-S fuzzy systems, each state is sampled at a different rate, causing the membership functions of the system and the controller to be evaluated at different sampling instants. Although such a mismatch also exists in single-rate systems, it becomes more severe under multi-rate sampling and cannot be neglected without introducing conservatism or instability. To address this, an effective relaxation method is proposed that explicitly exploits the structural properties of the FBFs mismatch in deriving the LMI-based stabilization conditions, providing a rigorous and systematic treatment of this problem for the first time in the multi-rate setting.

**Notations:** Throughout, $N_{n}$ is the set of positive integers {1,⋯,n}, $N_{0}$ is the set of nonnegative integers, and ${\mathbb{R}}^{n\times m}$ is the space of n×m real matrices. The symbol ⊗ denotes the Kronecker product, col{·} is the column vector, diag{·} is the block-diagonal matrix, $\left[a_{i}{]}_{n}=\text{diag}\{a_{1},a_{2},\cdots ,a_{n}\right\}$, $I_{n}$ is the *n*-dimension identity matrix, and $0_{n\times m}$ is the n×m zero matrix. For a square matrix R, $\text{He}\{R\}=R+R^{T}$, $R^{-1}$ indicates the inverse, $\lambda_{\max}(R)$ and $\lambda_{\min}(R)$ denote the maximum and minimum eigenvalues of R, respectively, and (*) denotes entries implied by symmetry in block-symmetric matrices. For compact representation of the LMIs, the vectors $e_{i}$ are defined as block-selection matrices that indicate the position of each block in the augmented vector.

## 2 System description and preliminaries

Let us consider the following continuous-time T-S fuzzy systems:


$$\begin{array}{c} \begin{array}{c} \left\{ \begin{array}{c} \dot{x}(t)=A(\theta (t))x(t)+B(\theta (t))u(t)+D(\theta (t))w(t)\\ z(t)=C(\theta (t))x(t) \end{array}\right. \end{array} \end{array}$$ (1)

subject to


$$\begin{array}{c} \left[\begin{array}{cccc} A(\theta (t)) & B(\theta (t)) & C(\theta (t)) & D(\theta (t)) \end{array}\right]=\sum_{i=1}^{r}\theta_{i}(t)\left[\begin{array}{cccc} A_{i} & B_{i} & C_{i} & D_{i} \end{array}\right] \end{array}$$

where $x(t)\in R^{n}$, $u(t)\in R^{m}$, $z(t)\in R^{n_{z}}$, and $w(t)\in R^{n_{x}}$ denote the system state, control input, performance output, and external disturbances, respectively; $A_{i}$, $B_{i}$, $C_{i}$, and $D_{i}$ are known system matrices; and $\theta (t)=\text{col}\{\theta_{1}(t),\cdots ,\theta_{r}(t)\}\in R^{r}$ indicates the FBF vector with *r* fuzzy rules. Particularly, the FBFs $\theta_{i}(t)$ satisfy that


$$\begin{array}{c} \sum_{i=1}^{r}\theta_{i}(t)=1,\;0\leq \theta_{i}(t)\leq 1,\;\forall i\in N_{r}. \end{array}$$ (2)

**Assumption 1.** Assume that FBF $\theta_{i}(t)$ is differentiable and satisfies


$$\begin{array}{c} |{\dot{\theta }}_{i}(t)|\leq \alpha_{i},\;\forall i\in N_{r}. \end{array}$$ (3)

**Assumption 2.** The external disturbance *w*(*t*) is assumed to be energy-bounded


$$\begin{array}{c} \int_{0}^{\infty }w^{T}(s)w(s)ds\leq \bar{w} \end{array}$$ (4)

where $\bar{w}$ is a known positive scalar.

**Fig 1:**
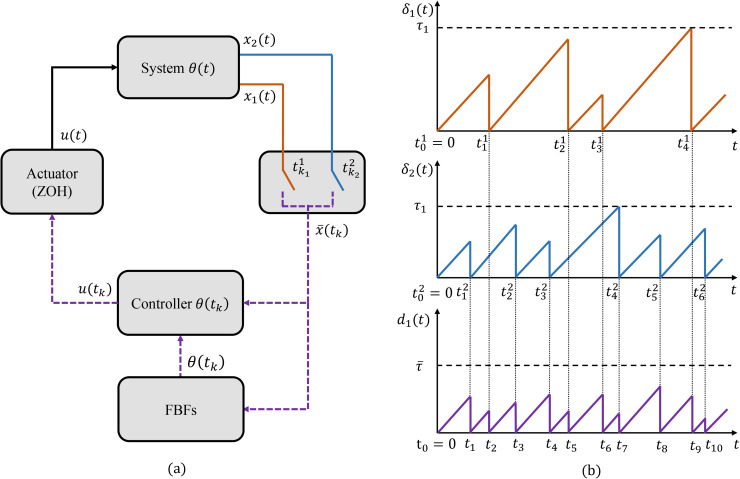
The multi-rate SDCSs framework: (a) illustration for the case *n* = 2, and (b) the sampling-induced delay $\delta_{1}(t)$, $\delta_{2}(t)$, and *d*(*t*).

Moreover, we consider a multi-rate sampling scenario in which each state component $x_{i}(t)$ is measured at its own sampling sequence $\{t_{k_{i}}^{i}{\}}_{k_{i}\geq 0}$, with $t_{0}^{i}=0$ and $t_{k_{i}}^{i}<t_{k_{i}+1}^{i}$ for all i∈{1,⋯,n} and $k_{i}\in N_{0}$. For illustration, Fig 1 depicts a diagram of multi-rate SDCS with *n* = 2. Accordingly, the global update times $\{t_{k}{\}}_{k\geq 0}$ collect all component-wise sampling instants


$$\begin{array}{c} \begin{array}{cc} & t_{0}=0,\;t_{k+1}=t_{k}+\min\{t_{k_{1}+1}^{1}-t_{k},\cdots ,t_{k_{n}+1}^{n}-t_{k}\} \end{array} \end{array}$$ (5)

$$\begin{array}{c} \begin{array}{cc} & h_{k}=t_{k+1}-t_{k},\;\underset{―}{\tau }\leq h_{k}\leq \bar{h}=\min\{\tau_{1},\cdots ,\tau_{n}\} \end{array} \end{array}$$ (6)

where $\underset{―}{\tau }$ denotes the minimum inter-sampling time of system and $\tau_{i}$ is the MASI of $x_{i}(t)$. In the considered sampling scheme, the actuator saturation control law is given as:


$$\begin{array}{c} u(t)=\text{sat}\{u(t_{k}),\bar{u}\},\;t\in [t_{k},t_{k+1}),\;\forall k\in N_{0} \end{array}$$ (7)

where $u(t_{k})$ is the sampled-data control input and $\bar{u}$ is the saturation level. The ℓ−th element of *u*(*t*) is given by


$$\begin{array}{c} u_{\ell }(t)=\text{sign}(u_{\ell }(t_{k}))\times \min\{u_{\ell }(t_{k}),{\bar{u}}_{\ell }\},\;\forall \ell \in N_{m}. \end{array}$$

Furthermore, according to [34, Theorem 1], there exists an auxiliary control input $v(t_{k})$ subject to $|v(t_{k})|\leq \bar{u}$, and (7) can be rewritten as follows:


$$\begin{array}{c} u(t)=\sum_{i=1}^{2^{m}}\phi_{i}(t_{k})(\Xi_{i}u(t_{k})+{\bar{\Xi }}_{i}v(t_{k})),\;t\in [t_{k},t_{k+1}) \end{array}$$ (8)

where $\phi_{i}(t_{k})$ is the weighting function satisfying


$$\begin{array}{c} \sum_{i=1}^{2^{m}}\phi_{i}(t_{k})=1,\;0\leq \phi_{i}(t_{k})\leq 1,\;\forall i\in N_{2^{m}} \end{array}$$ (9)

$\Xi_{i}$ and ${\bar{\Xi }}_{i}=I_{m}-\Xi_{i}$ are m×m diagonal matrices whose diagonal elements are either 1 or 0. Furthermore, the following control law is employed:


$$\begin{array}{c} u(t_{k})=F(\theta (t_{k}))\bar{x}(t_{k})\;\text{and}\;v(t_{k})=K(\theta (t_{k}))\bar{x}(t_{k}) \end{array}$$ (10)

where $\bar{x}(t_{k})=\text{col}\{x_{1}(t_{k_{1}}^{1}),\cdots ,x_{n}(t_{k_{n}}^{n})\}$ denotes sampled state, which is updated upon any component $x_{i}(t_{k_{i}}^{i})$ becomes available, $F(\theta (t_{k}))\in R^{m\times n}$ and $K(\theta (t_{k}))\in R^{m\times n}$ denote the control gains to be designed later, and $\theta (t_{k})$ is the sampled-data dependent FBF. Consequently, from (1) and (10), the resulting closed-loop system (CLS) is given by


$$\begin{array}{c} \begin{array}{c} \left\{ \begin{array}{c} \dot{x}(t)=A(\theta (t))x(t)+\sum_{\kappa =1}^{2^{m}}\phi_{\kappa }(t_{k}){\bar{B}}_{i}(\theta (t),\theta (t_{k}))x(t_{k})+E(\theta (t))w(t)\\ z(t)=C(\theta (t))x(t) \end{array}\right. \end{array} \end{array}$$ (11)

subject to


$$\begin{array}{c} x(t_{k})\in L(K(\theta (t_{k})),\bar{u})=\left\{x(t_{k})\in R^{n}\;|\;|K(\theta (t_{k}))x(t_{k})|\leq \bar{u}\right\} \end{array}$$ (12)

where ${\bar{B}}_{\kappa }(\theta (t),\theta (t_{k}))=B(\theta (t))(\Xi_{\kappa }F(\theta (t_{k}))+{\bar{\Xi }}_{\kappa }K(\theta (t_{k})))$. For the sake of brevity, we will use the following simplified notations: θ=θ(t), $\hat{\theta }=\theta (t_{k})$, and $\bar{\theta }=\hat{\theta }-\theta$. In addition, from (2) and (3), we derive the following constraints on the time derivatives of the FBFs and FBFs mismatch:


$$\begin{array}{c} \begin{array}{cc} & {\dot{\theta }}_{r}=-\sum_{i=1}^{r-1}{\dot{\theta }}_{i},\;|{\dot{\theta }}_{i}|\leq \alpha_{i},\;\forall i\in N_{r} \end{array} \end{array}$$ (13)

$$\begin{array}{c} \begin{array}{cc} & {\bar{\theta }}_{r}=-\sum_{i=1}^{r-1}{\bar{\theta }}_{i},\;|{\bar{\theta }}_{i}|\leq \beta_{i}=\min\{\bar{h}\alpha_{i},1\},\;\forall i\in N_{r}. \end{array} \end{array}$$ (14)

**Remark 1.** In practice, obtaining an accurate bound on the time derivative of the FBFs in advance is non-trivial, particularly when the control input is unconstrained. However, for a given system with a known operating region, the bound $\alpha_{i}$ in (13) can be derived analytically from the system dynamics. Specifically, since ${\dot{\theta }}_{i}(t)$ depends on the state and input through the premise variable, its bound can be estimated by separately bounding the gain and rate terms of ${\dot{\theta }}_{i}(t)$ over the prescribed operating region and admissible input set. A detailed derivation of $\alpha_{i}$ for the truck–trailer example is provided in the Illuminate examples section.

The following lemma will be used throughout to establish the main results.

**Lemma 1.1** ([35]). For given matrix $0<S=S^{T}\in R^{n\times n}$ and continuous function $\eta (t)\in R^{n}$ for all time t∈[a, b], the following inequality holds:


$$\begin{array}{c} \int_{a}^{b}\eta^{T}(s)S\eta (s)ds\geq \frac{1}{b-a}\left(\int_{a}^{b}\eta^{T}(s)ds\right)S\left(\int_{a}^{b}\eta^{T}(s)ds\right). \end{array}$$ (15)

**Lemma 1.2** ([36]). Let $g_{1},g_{2},\cdots ,g_{N}:R^{m}\mapsto R$ have positive values in an open subset D of $R^{m}$. Then, the reciprocally convex combination of $g_{\ell }$ over D satisfies


$$\begin{array}{c} \min_{\left\{\varepsilon_{\ell }|\varepsilon_{\ell }>0,\sum_{\ell }\varepsilon_{\ell }=1\right\}}\sum_{\ell }\frac{1}{\varepsilon_{\ell }}g_{\ell }(t)=\sum_{\ell }g_{\ell }(t)+\max_{f_{\ell \kappa }(t)}\sum_{\ell \text{≠}\kappa }f_{\ell \kappa }(t) \end{array}$$ (16)

subject to


$$\begin{array}{c} \left\{f_{\ell \kappa }:R^{m}\to R,f_{\kappa \ell }(t)\overset{△}{=}f_{\ell \kappa }(t),\left[\begin{array}{cc} g_{\ell }(t) & \left(*\right)\\ f_{\kappa \ell }(t) & g_{\kappa }(t) \end{array}\right]\geq 0\right\}. \end{array}$$

**Lemma 1.3** ([37]). For any matrix $\Omega_{ij}=\Omega_{ij}^{T}$, the condition $0>\sum_{i=1}^{r}\sum_{j=1}^{r}\theta_{i}\theta_{j}\Omega_{ij}$ subject to $\sum_{i=1}^{r}\theta_{i}(t)=1$ and $\theta_{i}(t)\in [0,1]$ holds, if it is satisfied that for all $i\in N_{r}$


$$\begin{array}{c} 0>\Omega_{ii},\;0>\Omega_{ii}+\sum_{j=1,j\text{≠}i}^{r}\frac{1}{2}(\Omega_{ij}+\Omega_{ji}). \end{array}$$ (17)

## 3 Control synthesis

This section derives stability and stabilization criteria for multi-rate SDCS T-S fuzzy systems. To reduce conservatism, we focus on exploiting the sampling-induced delay of each state $\delta_{i}(t)=t-t_{k_{i}}^{i}$, which satisfies $\delta_{i}(t)\in [0,\tau_{i}]$. For convenience, the following notation will be used throughout the remainder of the paper.


$$\begin{array}{cc} & d_{1}(t)=t-t_{k},\;d_{2}(t)=t_{k+1}-t,\;\delta (t)=\text{diag}\{\delta_{1}(t),\cdots ,\delta_{n}(t)\}\in R^{n\times n}\\ & \bar{\tau }=\max\{\tau_{1},\cdots ,\tau_{n}\},\;\Gamma =\text{diag}\{\tau_{1},\cdots ,\tau_{n}\}\in R^{n\times n}\\ & \tilde{x}(t)=\text{col}\{x_{1}(t-\tau_{1}),\cdots ,x_{n}(t-\tau_{n})\}\in R^{n} \end{array}$$

which imply $\dot{\delta }(t)=I_{n}$ and $0\leq \delta (t)\leq \bar{\tau }I_{n}$.

### 3.1 Stability analysis

This section aims to propose an improved stability analysis for multi-rate sampled-data linear systems, i.e., $\dot{x}(t)=Ax(t)+BF\bar{x}(t_{k})$. Let us establish the following LKF:


$$\begin{array}{c} V(t)=V_{1}(t)+V_{2}(t)+V_{3}(t)+V_{4}(t),\text{ for }t\in [t_{k},t_{k+1}) \end{array}$$ (18)

where


$$\begin{array}{cc} & V_{1}(t)=x^{T}(t)Px(t)\\ & V_{2}(t)=\sum_{i=1}^{n}\int_{t-\tau_{i}}^{t}x_{i}^{T}(s)S_{1i}x_{i}(s)ds+\sum_{i=1}^{n}\tau_{i}\int_{-\tau_{i}}^{0}\int_{t+\theta }^{t}{\dot{x}}_{i}^{T}(s)S_{2i}{\dot{x}}_{i}(s)dsd\theta \\ & V_{3}(t)=2d_{2}(t)\eta_{1}^{T}(t)W_{1}\eta_{2}(t)+2d_{1}(t)\eta_{3}^{T}(t)W_{2}\eta_{4}(t)\\ & V_{4}(t)=\eta_{5}^{T}(t)R_{1}\eta_{5}(t)+2(x(t)-\bar{x}(t_{k}){)}^{T}R_{2}\eta_{5}(t)+\sum_{i=1}^{n}\int_{t_{k_{i}}^{i}}^{t}{\dot{x}}_{i}^{T}(s)R_{6i}{\dot{x}}_{i}(s)ds \end{array}$$

where


$$\begin{array}{cc} & \eta (t)=\text{col}\left\{x(t),\delta (t)\bar{x}(t_{k}),\delta (t_{k})\bar{x}(t_{k}),\delta (t_{k+1})\bar{x}(t_{k}),\bar{x}(t_{k}),\tilde{x}(t)\right\}\in R^{6n}\\ & \eta_{1}(t)=\text{col}\left\{\delta (t)\bar{x}(t_{k})-\delta (t_{k})\bar{x}(t_{k}),d_{1}(t)\bar{x}(t_{k})\right\}\in R^{2n}\\ & \eta_{2}(t)=\text{col}\left\{\delta (t)\bar{x}(t_{k}),\delta (t_{k})\bar{x}(t_{k}),\bar{x}(t_{k})\right\}\in R^{3n}\\ & \eta_{3}(t)=\text{col}\left\{\delta (t_{k+1})\bar{x}(t_{k})-\delta (t)\bar{x}(t_{k}),d_{2}(t)\bar{x}(t_{k})\right\}\in R^{2n}\\ & \eta_{4}(t)=\text{col}\left\{\delta (t)\bar{x}(t_{k}),\delta (t_{k+1})\bar{x}(t_{k}),\bar{x}(t_{k})\right\}\in R^{3n},\;\eta_{5}(t)=\text{col}\left\{x(t),\bar{x}(t_{k})\right\}\in R^{2n} \end{array}$$

and $0<P=P^{T}\in R^{n\times n}$, $0<S_{1}=\text{diag}\{S_{1i},\cdots ,S_{1n}\}\in R^{n\times n}$, $0<S_{2}=\text{diag}\{S_{2i},\cdots ,S_{2n}\}\in R^{n\times n}$, $W_{1}\in R^{2n\times 3n}$, $W_{2}\in R^{2n\times 3n}$, $R_{\ell i}\in R$, for ℓ∈{1,2,⋯,6}, and


$$\begin{array}{cc} & R_{1}=\left[\begin{array}{cc} \bar{\tau }R_{1} & \left(*\right)\\ \bar{\tau }R_{2} & \bar{\tau }R_{3} \end{array}\right]=\left[\begin{array}{cc} \bar{\tau }{\left[R_{1i}\right]}_{n} & \left(*\right)\\ \bar{\tau }{\left[R_{2i}\right]}_{n} & \bar{\tau }{\left[R_{3i}\right]}_{n} \end{array}\right]\\ & R_{2}=\left[\begin{array}{cc} R_{4} & R_{5} \end{array}\right]=\left[\begin{array}{cc} {\left[R_{4i}\right]}_{n} & {\left[R_{5i}\right]}_{n} \end{array}\right],\;R_{6}={\left[R_{6i}\right]}_{n} \end{array}$$

subject to


$$\begin{array}{c} \begin{array}{cc} & 0<\left[\begin{array}{ccc} R_{1} & \left(*\right) & \left(*\right)\\ R_{2} & R_{3} & \left(*\right)\\ R_{4} & R_{5} & R_{6} \end{array}\right]. \end{array} \end{array}$$ (19)

In particular, *V*_1_(*t*) is the standard LKF and *V*_2_(*t*) is the time-dependent integral term capturing state-specific input-delay information. To reduce conservatism, we introduce two auxiliary terms: a looped functional *V*_3_(*t*) and a discontinuous functional *V*_4_(*t*). The looped functional *V*_3_(*t*) in (18) is designed to vanish at $t_{k}$ and $t_{k+1}$ so that it represents a virtual energy balance defined only within one sampling interval. In other words, the functional resets at each sampling instant, ensuring that no hidden energy is carried across consecutive sampling periods. This construction allows *V*_3_(*t*) to be not necessarily positive definite, thereby providing additional flexibility in *t*he stability analysis and resulting in less conservative conditions. Meanwhile, condition (19) guarantees


$$\begin{array}{cc} 0 & \leq \sum_{i=1}^{n}\int_{t_{k_{i}}^{i}}^{t}{\left[\begin{array}{c} x_{i}(t)\\ x_{i}(t_{k_{i}}^{i})\\ {\dot{x}}_{i}(s) \end{array}\right]}^{T}\left[\begin{array}{ccc} R_{1i} & \left(*\right) & \left(*\right)\\ R_{2i} & R_{3i} & \left(*\right)\\ R_{4i} & R_{5i} & R_{6i} \end{array}\right]\left[\begin{array}{c} x_{i}(t)\\ x_{i}(t_{k_{i}}^{i})\\ {\dot{x}}_{i}(s) \end{array}\right]ds\\ & \leq x^{T}(t)\tau R_{1}x(t)+2{\bar{x}}^{T}(t_{k})\tau R_{2}x(t)+{\bar{x}}^{T}(t_{k})\tau R_{3}\bar{x}(t_{k})\\ & \;+2(x^{T}(t)-{\bar{x}}^{T}(t_{k}))(R_{4}x(t)+R_{5}\bar{x}(t_{k}))+\sum_{i=1}^{n}\int_{t_{k_{i}}^{i}}^{t}{\dot{x}}_{i}^{T}(s)R_{6i}{\dot{x}}_{i}(s)ds. \end{array}$$ (20)

Therefore, $V_{4}(t)\geq 0$ for all $t\in [t_{k},t_{k+1})$. Moreover, since $\int_{t_{k_{i}}^{i}}^{t}{\dot{x}}_{i}^{T}(s)R_{6i}{\dot{x}}_{i}(s)ds$ vanishes at $t=t_{k_{i}}^{i}$, the jump of *V*_4_(*t*) at each sampling instant $t_{k_{i}}^{i}$ is diminished.

**Remark 2.** In contrast to [19–21], we introduces the delay-dependent terms $\delta (t)\bar{x}(t_{k})$, $\delta (t_{k})\bar{x}(t_{k})$, and $\delta (t_{k+1})\bar{x}(t_{k})$ into looped-functional *V*_3_(*t*). Incorporating these sampling-driven terms exploits additional information about per-state sampling delays, resulting in less conservative s*t*ability and stabilization conditions. In addition, studies in [20] and [21] term involving the state at the controller-reception instant, i.e., $x(t_{k})$ and $\int_{t_{k}}^{t}x(s)\;ds$, which is based on a single rate sampling and is not suitable for MRSD settings. By reconstructing the looped-functional to focus solely on the relevant state components in a multi-rate sampling scenario, the proposed method achieves better performance while maintaining lower computational complexity than existing approaches.

**Remark 3.** The discontinuous function *V*_4_(*t*) is constructed for multi-rate sampling scenarios so that it captures the relationship between the current state and the most recent sampled state of each component, i.e., *x*(*t*) and $\bar{x}(t_{k})$. In addition, introducing a free-weighting matrix *R* into the stability and stabilization conditions helps reduce conservatism in the derived results.

**Lemma 2.1.** For given positive scalars σ, $\underset{―}{\tau }$, and $\tau_{i}$, for $i\in N_{n}$, CLS (11) is asymptotically stable if there exist matrices $0<P=P^{T}\in R^{n\times n}$, $0<S_{1}=\text{diag}\{S_{1i},\cdots ,S_{1n}\}\in R^{n\times n}$, $0<S_{2}=\text{diag}\{S_{2i},\cdots ,S_{2n}\}\in R^{n\times n}$, $R_{\ell }=\text{diag}\{R_{\ell i},\cdots ,R_{\ell n}\}\in R^{n\times n}$, for ℓ∈{1,2,⋯,6}, $M=\text{diag}\{M_{i},\cdots ,M_{n}\}\in R^{n\times n}$, $W_{1}\in R^{2n\times 3n}$, and $W_{2}\in R^{2n\times 3n}$, $N_{1}\in R^{n\times 2n}$, $N_{2}\in R^{n\times 2n}$, $N_{3}\in R^{n\times 2n}$, and $0<U=U^{T}\in R^{n\times n}$, such that the following conditions are satisfied, for $h_{k}\in \{\underset{―}{\tau },\bar{h}\}:$ LMI (19) and


$$\begin{array}{c} \begin{array}{cc} & 0<\left[\begin{array}{cc} S_{2} & \left(*\right)\\ M & S_{2} \end{array}\right] \end{array} \end{array}$$ (21)

$$\begin{array}{c} \begin{array}{cc} & 0>\left[\begin{array}{cc} \Phi_{0}(t_{k})+h_{k}\Phi_{1}(t_{k}) & \left(*\right)\\ \bar{\tau }(N_{1}\Xi_{5}+N_{2}\Xi_{6}+N_{3}\Xi_{7}) & -U \end{array}\right] \end{array} \end{array}$$ (22)

$$\begin{array}{c} \begin{array}{cc} & 0>\left[\begin{array}{cc} \Phi_{0}(t_{k+1})+h_{k}\Phi_{2}(t_{k+1}) & \left(*\right)\\ \bar{\tau }(N_{1}\Xi_{5}+N_{2}\Xi_{6}+N_{3}\Xi_{7}) & -U \end{array}\right] \end{array} \end{array}$$ (23)

where


$$\begin{array}{cc} & \Phi_{0}(t)=e_{1}^{T}\sigma Pe_{1}+\text{He}\{e_{1}^{T}P\Xi_{0}\}+e_{1}^{T}S_{1}e_{1}-e_{6}^{T}S_{1}e_{6}+\Xi_{0}^{T}\Gamma S_{2}\Gamma \Xi_{0}\\ & \;-(e_{1}^{T}-e_{5}^{T})S_{2}(e_{1}-e_{5})-(e_{5}^{T}-e_{6}^{T})S_{2}(e_{5}-e_{6})+e_{5}^{T}Ue_{5}+\Xi_{0}^{T}R_{6}\Xi_{0}\\ & \;+\text{He}\{-\Xi_{1}^{T}(t)W_{1}\Xi_{2}+\Xi_{3}^{T}(t)W_{2}\Xi_{4}+\Xi_{0}^{T}\bar{\tau }R_{1}e_{1}+e_{5}^{T}R_{2}\bar{\tau }\Xi_{0}\}\\ & \;+\text{He}\{(e_{1}^{T}-e_{5}^{T})R_{4}\Xi_{0}+\Xi_{0}^{T}R_{4}e_{1}+\Xi_{0}^{T}R_{5}e_{5}-(e_{5}^{T}-e_{6}^{T})M(e_{1}-e_{5})\}\\ & \;-\text{He}\{e_{2}^{T}N_{1}\Xi_{5}+e_{3}^{T}N_{2}\Xi_{6}+e_{4}^{T}N_{3}\Xi_{7}\}\\ & \Phi_{1}(t_{k})=\text{He}\{\Xi_{1d}^{T}W_{1}\Xi_{2}+\Xi_{1}^{T}(t_{k})W_{1}\Xi_{2d}+e_{5}^{T}N_{3}\Xi_{7}\}\\ & \Phi_{2}(t_{k+1})=\text{He}\{\Xi_{3d}^{T}W_{2}\Xi_{4}+\Xi_{3}^{T}(t_{k+1})W_{2}\Xi_{4d}-e_{5}^{T}N_{2}\Xi_{6}\} \end{array}$$

in which


$$\begin{array}{cc} & e_{p}=\left[\begin{array}{ccc} 0_{n\times (p-1)n} & I_{n} & 0_{n\times (6-p)n} \end{array}\right]\in R^{n\times 6n}\\ & \Xi_{0}=Ae_{1}+BFe_{5},\;\Xi_{1}(t)=\text{col}\{e_{2}-e_{3},d_{1}(t)e_{5}\},\;\Xi_{1d}=\text{col}\{e_{5},e_{5}\}\\ & \Xi_{2}=\text{col}\{e_{2},e_{3},e_{5}\},\;\Xi_{2d}=\text{col}\{e_{5},0,0\},\;\Xi_{3}(t)=\text{col}\{e_{4}-e_{2},d_{2}(t)e_{5}\}\\ & \Xi_{3d}=\text{col}\{-e_{5},-e_{5}\},\;\Xi_{4}=\text{col}\{e_{2},e_{4},e_{5}\},\;\Xi_{4d}=\text{col}\{e_{5},0,0\}\\ & \Xi_{5}=\text{col}\{e_{2},e_{5}\},\;\Xi_{6}=\text{col}\{e_{3},e_{5}\},\;\Xi_{7}=\text{col}\{e_{4},e_{5}\}. \end{array}$$

*Proof:* The time derivatives of (18) are given by:


$$\begin{array}{c} \begin{array}{cc} & {\dot{V}}_{1}(t)=2x^{T}(t)P\dot{x}(t) \end{array} \end{array}$$ (24)

$$\begin{array}{c} \begin{array}{cc} & {\dot{V}}_{2}(t)=\sum_{i=1}^{n}x_{i}^{T}(t)S_{1i}x_{i}(t)-\sum_{i=1}^{n}(x_{i}^{T}(t-\tau_{i})S_{1i}x_{i}(t-\tau_{i})-\tau_{i}^{2}{\dot{x}}_{i}^{T}(t)S_{2i}{\dot{x}}_{i}(t))\\ & \;{\underset{―}{-\sum_{i=1}^{n}\tau_{i}\int_{t-\tau_{i}}^{t}{\dot{x}}_{i}^{T}(s)S_{2i}{\dot{x}}_{i}(s)ds}}_{:=T(t)}\\ & \;\leq x^{T}(t)S_{1}x(t)-{\tilde{x}}^{T}(t)S_{1}\tilde{x}(t)+{\dot{x}}^{T}(t)\Gamma S_{2}\Gamma \dot{x}(t)+T_{1}(t) \end{array} \end{array}$$ (25)

$$\begin{array}{c} \begin{array}{cc} & {\dot{V}}_{3}(t)=-2\eta_{1}^{T}(t)W_{1}\eta_{2}(t)+2d_{2}(t)({\dot{\eta }}_{1}^{T}(t)W_{1}\eta_{2}(t)+\eta_{1}^{T}(t)W_{1}{\dot{\eta }}_{2}(t))\\ & \;+2\eta_{3}^{T}(t)W_{2}\eta_{4}(t)+2d_{1}(t)(2{\dot{\eta }}_{3}^{T}(t)W_{2}\eta_{4}(t)+2\eta_{3}^{T}(t)W_{2}{\dot{\eta }}_{4}(t)) \end{array} \end{array}$$ (26)

$$\begin{array}{c} \begin{array}{cc} & {\dot{V}}_{4}(t)=2{\dot{\eta }}_{3}(t)R_{1}\eta_{3}(t)+2{\dot{x}}^{T}(t)R_{2}\eta_{3}(t)+2(x^{T}(t)-{\bar{x}}^{T}(t_{k}))R_{2}{\dot{\eta }}_{3}(t)+\sum_{i=1}^{n}{\dot{x}}_{i}^{T}(t)R_{6i}{\dot{x}}_{i}(t)\\ & \;=2{\dot{\eta }}_{3}(t)R_{1}\eta_{3}(t)+2{\dot{x}}^{T}(t)R_{2}\eta_{3}(t)+2(x^{T}(t)-{\bar{x}}^{T}(t_{k}))R_{2}{\dot{\eta }}_{3}(t)+{\dot{x}}^{T}(t)R_{6}\dot{x}(t). \end{array} \end{array}$$ (27)

From Lemma 1.1, it follows that


$$\begin{array}{c} \begin{array}{cc} T_{1}(t) & =-\sum_{i=1}^{n}\tau_{i}\int_{t_{k_{i}}^{i}}^{t}{\dot{x}}_{i}^{T}(s)S_{2i}{\dot{x}}_{i}(s)ds-\sum_{i=1}^{n}\tau_{i}\int_{t-\tau_{i}}^{t_{k_{i}}^{i}}{\dot{x}}_{i}^{T}(s)S_{2i}{\dot{x}}_{i}(s)ds\\ & \;\leq -\sum_{i=1}^{n}\frac{\tau_{i}}{\delta_{i}(t)}(x_{i}(t)-x_{i}(t_{k_{i}}^{i}){)}^{T}S_{2i}(x_{i}(t)-x_{i}(t_{k_{i}}^{i}))\\ & \;-\sum_{i=1}^{n}\frac{\tau_{i}}{\tau_{i}-\delta_{i}(t)}(x_{i}(t_{k_{i}}^{i})-x_{i}(t-\tau_{i}){)}^{T}S_{2i}(x_{i}(t_{k_{i}}^{i})-x_{i}(t-\tau_{i})). \end{array} \end{array}$$ (28)

Then, applying Lemma 1.2 and choosing


$$\begin{array}{cc} & \varepsilon_{1i}=\frac{\delta_{i}(t)}{\tau_{i}},\;g_{1i}(t)=(x_{i}(t)-x_{i}(t_{k_{i}}^{i}){)}^{T}S_{2i}(x_{i}(t)-x_{i}(t_{k_{i}}^{i}))\\ & \varepsilon_{2i}=\frac{\tau_{i}-\delta_{i}(t)}{\tau_{i}},\;g_{2i}(t)=(x_{i}(t_{k_{i}}^{i})-x_{i}(t-\tau_{i}){)}^{T}S_{2i}(x_{i}(t_{k_{i}}^{i})-x_{i}(t-\tau_{i}))\\ & f_{21i}(t)=(x_{i}(t_{k_{i}}^{i})-x_{i}(t-\tau_{i}){)}^{T}M_{i}(x_{i}(t)-x_{i}(t_{k_{i}}^{i})) \end{array}$$

condition (28) subject to (21), can be reformulated as:


$$\begin{array}{c} \begin{array}{cc} T_{1}(t) & \leq -\sum_{i=1}^{n}{\left[\begin{array}{c} x_{i}(t)-x_{i}(t_{k_{i}}^{i})\\ x_{i}(t_{k_{i}}^{i})-x_{i}(t-\tau_{i}) \end{array}\right]}^{T}\left[\begin{array}{cc} S_{2i} & \left(*\right)\\ M_{i} & S_{2i} \end{array}\right]\left[\begin{array}{c} x_{i}(t)-x_{i}(t_{k_{i}}^{i})\\ x_{i}(t_{k_{i}}^{i})-x_{i}(t-\tau_{i}) \end{array}\right]\\ & =-{\left[\begin{array}{c} x(t)-\bar{x}(t_{k})\\ \bar{x}(t_{k})-\tilde{x}(t) \end{array}\right]}^{T}\left[\begin{array}{cc} S_{2} & \left(*\right)\\ M & S_{2} \end{array}\right]\left[\begin{array}{c} x(t)-\bar{x}(t_{k})\\ \bar{x}(t_{k})-\tilde{x}(t) \end{array}\right]. \end{array} \end{array}$$ (29)

Moreover, the following zero equalities are used as auxiliary relations:


$$\begin{array}{cc} 0 & =2\eta^{T}(t)(e_{5}^{T}\delta (t)-e_{2}^{T})N_{1}\Xi_{5}\eta (t)\\ 0 & =2\eta^{T}(t)(e_{5}^{T}\delta (t_{k})-e_{3}^{T})N_{2}\Xi_{6}\eta (t)\\ 0 & =2\eta^{T}(t)(e_{5}^{T}\delta (t_{k+1})-e_{4}^{T})N_{3}\Xi_{7}\eta (t) \end{array}$$

which are equivalent to


$$\begin{array}{c} \begin{array}{cc} 0 & =-2\eta^{T}(t)(e_{2}^{T}N_{1}\Xi_{5}+e_{3}^{T}N_{2}\Xi_{6}+e_{4}^{T}N_{3}\Xi_{7}-d_{1}(t)e_{5}^{T}N_{2}\Xi_{6}+d_{2}(t)e_{5}^{T}N_{3}\Xi_{7})\eta (t)\\ & \;{\underset{―}{+\;2\eta^{T}(t)e_{5}^{T}\delta (t)(N_{1}\Xi_{5}+N_{2}\Xi_{6}+N_{3}\Xi_{7})\eta (t)}}_{:=T_{2}(t)}. \end{array} \end{array}$$ (30)

Then, since $\delta (t)\leq \bar{\tau }I_{n}$, it follows from that


$$\begin{array}{c} T_{2}(t)\leq \eta^{T}(t)(e_{5}^{T}Ue_{5}+(N_{1}\Xi_{5}+N_{2}\Xi_{6}+N_{3}\Xi_{7}{)}^{T}{\bar{\tau }}^{2}U^{-1}(N_{1}\Xi_{5}+N_{2}\Xi_{6}+N_{3}\Xi_{7}))\eta (t). \end{array}$$ (31)

Consequently, from (24)-(26) together with (29)-(31), we obtain:


$$\begin{array}{c} \dot{V}(t)+\sigma V_{1}(t)\leq \eta^{T}(t)\Psi (t)\eta (t) \end{array}$$ (32)

where


$$\begin{array}{cc} \Psi (t) & =\Phi_{0}(t)+\frac{d_{2}(t)}{h_{k}}h_{k}\Phi_{1}(t)+\frac{d_{1}(t)}{h_{k}}h_{k}\Phi_{2}(t)\\ & \;+(N_{1}\Xi_{5}+N_{2}\Xi_{6}+N_{3}\Xi_{7}{)}^{T}{\bar{\tau }}^{2}U^{-1}(N_{1}\Xi_{5}+N_{2}\Xi_{6}+N_{3}\Xi_{7}). \end{array}$$

Since $\frac{d_{1}(t)+d_{2}(t)}{h_{k}}=1$, for $t\in [t_{k},\;t_{k+1}]$, the stability condition $\dot{V}(t)+\sigma V_{1}(t)<0$ (i.e., Ψ(t)<0) reduces to the convex combination at the endpoints, for $h_{k}\in \{\underset{―}{\tau },\;\bar{h}\}$:


$$\begin{array}{c} \begin{array}{cc} & 0>\Phi_{0}(t_{k})+h_{k}\Phi_{1}(t_{k})+(N_{1}\Xi_{5}+N_{2}\Xi_{6}+N_{3}\Xi_{7}{)}^{T}{\bar{\tau }}^{2}U^{-1}(N_{1}\Xi_{5}+N_{2}\Xi_{6}+N_{3}) \end{array} \end{array}$$ (33)

$$\begin{array}{c} \begin{array}{cc} & 0>\Phi_{0}(t_{k+1})+h_{k}\Phi_{2}(t_{k+1})+(N_{1}\Xi_{5}+N_{2}\Xi_{6}+N_{3}\Xi_{7}{)}^{T}{\bar{\tau }}^{2}U^{-1}(N_{1}\Xi_{5}+N_{2}\Xi_{6}+N_{3}) \end{array} \end{array}$$ (34)

which are transformed into (22) and (23) by Schur complement [38]. ■

**Remark 4.** To reduce conservatism, the slack matrices *N*_1_, *N*_2_, and *N*_3_ are introduced via the zero equality (30). Furthermore, the correlation between the current sampling-induced delay δ(t) and the maximum sampling interval $\bar{\tau }$ is incorporated into (31) to better exploit the sampling characteristics of the multi-rate systems.

**Remark 5.** In particular, the computational complexity of an LMI optimization problem can be roughly estimated as being proportional to $N_{var}^{3}N_{row}$, where $N_{var}$ denotes the total number of scalar decision variables and $N_{row}$ denotes the total row dimension of the LMIs (see [39]). For a given control gain, the LMI conditions in Lemma 2.1 require $\left(N_{var},N_{row})=(19n^{2}+10n,19n\right)$. In comparison, the methods in [20, Theorem 1] and [21, Theorem 1] require $\left(N_{var},N_{row})=(73.5n^{2}+6n,19n\right)$ and $\left(N_{var},N_{row})=(57.25n^{2}+5.5n,23n\right)$, respectively. Therefore, it can be observed that the proposed approach achieves a significantly reduced computational complexity.

### 3.2 Controller design

The following lemma establishes a basic stability condition for the CLS (11) with actuator saturation and external disturbances.

**Lemma 2.2.** For given positive scalars σ, γ, $\bar{w}$, and $\bar{u}$, CLS (11) subject to (12) is asymptotically stable with a guaranteed performance γ if the following conditions hold


$$\begin{array}{c} \begin{array}{cc} & 0\leq \left[\begin{array}{cc} P(\hat{\theta }) & \left(*\right)\\ \Lambda_{\ell }K(\hat{\theta }) & \nu_{\ell } \end{array}\right],\;\forall \ell \in N_{m} \end{array} \end{array}$$ (35)

$$\begin{array}{c} \begin{array}{cc} & 0>\left[\begin{array}{ccc} \Psi (\theta ,\hat{\theta },\dot{\theta }) & \left(*\right) & \left(*\right)\\ D^{T}(\theta )P(\theta ) & -\gamma^{2}I & 0\\ C(\theta ) & 0 & -I \end{array}\right] \end{array} \end{array}$$ (36)

where $\Lambda_{\ell }=\left[\begin{array}{ccc} 0_{1\times (\ell -1)} & 1 & 0_{1\times (m-\ell )} \end{array}\right]$ and $\nu_{\ell }=\frac{{\bar{u}}_{\ell }^{2}}{1+\frac{\gamma^{2}}{\sigma }{\bar{w}}^{2}}$.

*Proof:* Let us consider the following stability condition:


$$\begin{array}{c} 0>\dot{V}(t)+\sigma V(t)+\|z(t){\|}_{2}^{2}-\gamma^{2}\|w(t){\|}_{2}^{2}. \end{array}$$ (37)

Multiplying (37) by $e^{\sigma t}$ and integrating over $\left[t_{k-1},t_{k}\right)$ yields


$$\begin{array}{c} V(t_{k})<e^{\sigma (t_{k-1}-t_{k})}V(t_{k-1})-\int_{t_{k-1}}^{t_{k}}\;\;\;e^{\sigma (s-t_{k})}{\left\|z(s)\right\|}_{2}^{2}ds+\gamma^{2}\int_{t_{k-1}}^{t_{k}}\;\;\;e^{\sigma (s-t_{k})}{\left\|w(s)\right\|}^{2}ds. \end{array}$$ (38)

By recursively applying the above inequality from $t_{k}$ to *t*_0_, we obtain


$$\begin{array}{c} V(t_{k})<e^{-\sigma t_{k}}V(t_{0})-\int_{t_{0}}^{t_{k}}e^{\sigma (s-t_{k})}{\left\|z(s)\right\|}_{2}^{2}ds+\gamma^{2}\int_{t_{0}}^{t_{k}}e^{\sigma (s-t_{k})}{\left\|w(s)\right\|}_{2}^{2}ds. \end{array}$$ (39)

Hence, in the absence of disturbance, i.e., w(t)≡0, (39) yields


$$\begin{array}{c} \|x(t_{k}){\|}_{2}^{2}<e^{-\sigma t_{k}}\frac{\lambda_{\max}(P(\theta ))}{\lambda_{\min}(P(\theta ))}\|x(t_{0}){\|}_{2}^{2},\;\forall k\in N_{0} \end{array}$$ (40)

which means that system (11) is asymptotically stable. Next, consider an initial condition $x(t_{0})\in ℰ(P(\theta ),1)$. Based on Assumption 2, it follows from (39) that


$$\begin{array}{c} \begin{array}{cc} V(t_{k}) & <e^{-\sigma t_{k}}V(t_{0})+\gamma^{2}\int_{t_{0}}^{t_{k}}e^{\sigma (s-t_{k})}{\left\|w(s)\right\|}_{2}^{2}ds\\ & \leq 1+\frac{\gamma^{2}}{\sigma }{\bar{w}}^{2},\;\forall k\in N_{0}. \end{array} \end{array}$$ (41)

Then, by defining the ellipsoidal set


$$\begin{array}{c} E\left(P(\hat{\theta }),1+\frac{\gamma^{2}}{\sigma }{\bar{w}}^{2}\right)=\left\{x(t_{k})\in R^{n}\;|\;x^{T}(t_{k})P(\hat{\theta })x(t_{k})\leq 1+\frac{\gamma^{2}}{\sigma }{\bar{w}}^{2}\right\} \end{array}$$ (42)

the Schur complement of (35) yields


$$\begin{array}{c} \frac{1}{1+\frac{\gamma^{2}}{\sigma }{\bar{w}}^{2}}P(\hat{\theta })\geq \frac{1}{{\bar{u}}_{\ell }^{2}}K^{T}(\hat{\theta })\Lambda_{\ell }^{T}{\bar{\Lambda }}_{\ell }K(\hat{\theta }),\;\forall \ell \in N_{m} \end{array}$$

which implies the actuator saturation condition


$$\begin{array}{c} x(t_{k})\in E\left(P(\hat{\theta }),1+\frac{\gamma^{2}}{\sigma }{\bar{w}}^{2}\right)\subset L(K(\hat{\theta }),\bar{u}). \end{array}$$ (43)

Finally, with zero initial condition, i.e., $x(t_{0})\equiv 0$, it follows from (39) that


$$\begin{array}{c} \int_{t_{0}}^{t_{k}}{\left\|z(s)\right\|}_{2}^{2}ds<\gamma^{2}\int_{t_{0}}^{t_{k}}{\left\|w(s)\right\|}_{2}^{2}ds,\;\forall k\in N_{0}. \end{array}$$ (44)

which guarantees the ${ℋ}_{\infty }$ disturbance attenuation condition [4]. Consequently, recalling (37), we have


$$\begin{array}{c} \dot{V}(t)+\sigma V(t)+\|z(t){\|}_{2}^{2}-\gamma^{2}\|w(t){\|}_{2}^{2}\leq \eta^{*T}(t)\Psi^{*}(t)\eta^{*}(t) \end{array}$$ (45)

where $\eta^{*}(t)=\text{col}\{x(t),w(t)\}\in R^{n+n_{w}}$ and


$$\begin{array}{cc} & \Psi^{*}(t)=\left[\begin{array}{cc} \Psi (\theta ,\hat{\theta },\dot{\theta }) & \left(*\right)\\ D^{T}(\theta )P(\theta ) & -\gamma^{2}I \end{array}\right]+\left[\begin{array}{c} C^{T}(\theta )\\ 0 \end{array}\right]\left[\begin{array}{cc} C(\theta ) & 0 \end{array}\right] \end{array}$$

which is converted into (36) by the Schur complement. ■

Building on Lemma 2.1 and Lemma 2.2, the following lemma presents the PLMIs-based stabilization criterion for synthesizing the controller gain of the CLS (11).

**Lemma 2.3.** For given positive scalars σ, γ, $\bar{w}$, $\bar{u}$, $\underset{―}{\tau }$, and $\tau_{i}$, for $i\in N_{n}$, CLS (11) is asymptotically stable if there exist matrices $0<\bar{P}(\theta )=\bar{P}(\theta {)}^{T}\in R^{n\times n}$, $0<{\bar{S}}_{1}={\bar{S}}_{1}^{T}\in R^{n\times n}$, $0<{\bar{S}}_{2}={\bar{S}}_{2}^{T}\in R^{n\times n}$, ${\bar{R}}_{\ell }={\bar{R}}_{\ell }^{T}\in R^{n\times n}$, for ℓ∈{1,2,⋯,6}, $\bar{M}={\bar{M}}^{T}\in R^{n\times n}$, ${\bar{W}}_{1}\in R^{2n\times 3n}$, and ${\bar{W}}_{2}\in R^{2n\times 3n}$, ${\bar{N}}_{1}\in R^{n\times 2n}$, ${\bar{N}}_{2}\in R^{n\times 2n}$, ${\bar{N}}_{3}\in R^{n\times 2n}$, $0<\bar{U}={\bar{U}}^{T}\in R^{n\times n}$, $0<\bar{G}\in R^{n\times n}$, $\bar{F}(\hat{\theta })\in R^{m\times n}$ and $\bar{K}(\hat{\theta })\in R^{m\times n}$, such that the following conditions are satisfied, for all $\kappa \in N_{2^{m}}$, $\ell \in N_{m}$, $t\in \{t_{k},t_{k+1}\}$, and $h_{k}\in \{\underset{―}{\tau },\bar{h}\}:$


$$\begin{array}{c} \begin{array}{cc} & 0<\left[\begin{array}{ccc} {\bar{R}}_{1} & \left(*\right) & \left(*\right)\\ {\bar{R}}_{2} & {\bar{R}}_{3} & \left(*\right)\\ {\bar{R}}_{4} & {\bar{R}}_{5} & {\bar{R}}_{6} \end{array}\right] \end{array} \end{array}$$ (46)

$$\begin{array}{c} \begin{array}{cc} & 0<\left[\begin{array}{cc} {\bar{S}}_{2} & \left(*\right)\\ \bar{M} & {\bar{S}}_{2} \end{array}\right] \end{array} \end{array}$$ (47)

$$\begin{array}{c} \begin{array}{cc} & 0\leq \left[\begin{array}{cc} \bar{P}(\hat{\theta }) & \left(*\right)\\ \Lambda_{\ell }\bar{K}(\hat{\theta }) & \nu_{\ell } \end{array}\right] \end{array} \end{array}$$ (48)

$$\begin{array}{c} \begin{array}{cc} & 0>\left[\begin{array}{cccc} {\bar{\Phi }}_{\kappa }(\dot{\theta },\bar{\theta })+{\bar{\Phi }}_{\kappa }(\theta )+{\bar{\Phi }}_{0}(t)+h_{k}{\bar{\Phi }}_{t} & \left(*\right) & \left(*\right) & \left(*\right)\\ D^{T}(\theta )\Xi_{0} & -\gamma^{2}I & 0 & 0\\ C(\theta )\bar{G}e_{1} & 0 & -I & 0\\ \bar{\tau }({\bar{N}}_{1}\Xi_{5}+{\bar{N}}_{2}\Xi_{6}+{\bar{N}}_{3}\Xi_{7}) & 0 & 0 & -\bar{U} \end{array}\right] \end{array} \end{array}$$ (49)

where


$$\begin{array}{cc} & {\bar{\Phi }}_{\kappa }(\dot{\theta },\bar{\theta })=\text{He}\{e_{1}^{T}\dot{\bar{P}}(\theta )e_{1}+\Xi_{0}^{T}B(\theta )(\Xi_{\kappa }\bar{F}(\bar{\theta })+{\bar{\Xi }}_{\kappa }\bar{K}(\bar{\theta }))e_{5}\}\\ & {\bar{\Phi }}_{\kappa }(\theta )=e_{1}^{T}\sigma \bar{P}(\theta )e_{1}+\text{He}\{e_{1}^{T}\bar{P}(\theta )e_{7}+\Xi_{0}^{T}(A(\theta )\bar{G}e_{1}+B(\theta )(\Xi_{\kappa }\bar{F}(\theta )+{\bar{\Xi }}_{\kappa }\bar{K}(\theta ))e_{5})\}\\ & {\bar{\Phi }}_{0}(t)=e_{1}^{T}{\bar{S}}_{1}e_{1}-e_{6}^{T}{\bar{S}}_{1}e_{6}+e_{7}^{T}{\bar{\tau }}^{2}{\bar{S}}_{2}e_{7}-(e_{1}^{T}-e_{5}^{T}){\bar{S}}_{2}(e_{1}-e_{5})\\ & \;-(e_{5}^{T}-e_{6}^{T}){\bar{S}}_{2}(e_{5}-e_{6})+e_{5}^{T}\bar{U}e_{5}+e_{7}^{T}{\bar{R}}_{6}e_{7}-\text{He}\{\Xi_{0}^{T}\bar{G}e_{7}\}\\ & \;+\text{He}\{-\Xi_{1}^{T}(t){\bar{W}}_{1}\Xi_{2}+\Xi_{3}^{T}(t){\bar{W}}_{2}\Xi_{4}+e_{7}^{T}\bar{\tau }{\bar{R}}_{1}e_{1}+e_{5}^{T}\bar{\tau }{\bar{R}}_{2}e_{7}\}\\ & \;+\text{He}\{(e_{1}^{T}-e_{5}^{T}){\bar{R}}_{4}e_{7}+e_{7}^{T}{\bar{R}}_{4}e_{1}+e_{7}^{T}{\bar{R}}_{5}e_{5}-(e_{5}^{T}-e_{6}^{T})\bar{M}(e_{1}-e_{5})\}\\ & \;-\text{He}\{e_{2}^{T}{\bar{N}}_{1}\Xi_{5}+e_{3}^{T}{\bar{N}}_{2}\Xi_{6}+e_{4}^{T}{\bar{N}}_{3}\Xi_{7}\}\\ & {\bar{\Phi }}_{t_{k}}=\text{He}\{\Xi_{1d}^{T}{\bar{W}}_{1}\Xi_{2}+\Xi_{1}^{T}(t_{k}){\bar{W}}_{1}\Xi_{2d}+e_{5}^{T}{\bar{N}}_{3}\Xi_{7}\}\\ & {\bar{\Phi }}_{t_{k+1}}=\text{He}\{\Xi_{3d}^{T}{\bar{W}}_{2}\Xi_{4}+\Xi_{3}^{T}(t_{k+1}){\bar{W}}_{2}\Xi_{4d}-e_{5}^{T}{\bar{N}}_{2}\Xi_{6}\} \end{array}$$

in which


$$\begin{array}{cc} & \bar{F}(\hat{\theta })=\bar{F}(\bar{\theta })+\bar{F}(\theta ),\;e_{p}=\left[\begin{array}{ccc} 0_{n\times (p-1)n} & I_{n} & 0_{n\times (7-p)n} \end{array}\right]\in R^{n\times 7n}\\ & \Xi_{0}=\lambda_{1}e_{1}+\lambda_{2}e_{5}+e_{7},\;\Xi_{1}(t)=\text{col}\{e_{2}-e_{3},d_{1}(t)e_{5}\},\;\Xi_{1d}=\text{col}\{e_{5},e_{5}\}\\ & \Xi_{2}=\text{col}\{e_{2},e_{3},e_{5}\},\;\Xi_{2d}=\text{col}\{e_{5},0,0\},\;\Xi_{3}(t)=\text{col}\{e_{4}-e_{2},d_{2}(t)e_{5}\}\\ & \Xi_{3d}=\text{col}\{-e_{5},-e_{5}\},\;\Xi_{4}=\text{col}\{e_{2},e_{4},e_{5}\},\;\Xi_{4d}=\text{col}\{e_{5},0,0\}\\ & \Xi_{5}=\text{col}\{e_{2},e_{5}\},\Xi_{6}=\text{col}\{e_{3},e_{5}\},\Xi_{7}=\text{col}\{e_{4},e_{5}\}. \end{array}$$

Then, the control gain is obtained as $F(\hat{\theta })=\bar{F}(\hat{\theta }){\bar{G}}^{-1}$.

**Proof:** Let us define the FBF-dependent LKF


$$\begin{array}{c} V_{1}(t)=x^{T}(t)P(\theta )x(t) \end{array}$$ (50)

whose time derivative is given by


$$\begin{array}{c} {\dot{V}}_{1}(t)=2x^{T}(t)P(\theta )\dot{x}(t)+x^{T}(t)\dot{P}(\theta )x(t). \end{array}$$ (51)

In addition, it follows from (11) that


$$\begin{array}{c} \begin{array}{cc} 0 & =2(\lambda_{1}x^{T}(t)+\lambda_{2}{\bar{x}}^{T}(t_{k})+{\dot{x}}^{T}(t))G^{T}(A(\theta )x(t)+\sum_{\kappa =1}^{2^{m}}\phi_{\kappa }(t_{k}){\bar{B}}_{\kappa }(\theta ,\hat{\theta })\bar{x}(t_{k})-\dot{x}(t))\\ & \;+2(\lambda_{1}x^{T}(t)+\lambda_{2}{\bar{x}}^{T}(t_{k})+{\dot{x}}^{T}(t))G^{T}D(\theta )w(t). \end{array} \end{array}$$ (52)

Then, we define the additional augmented state $\bar{\eta }(t)=\text{col}\{\eta (t),\;\dot{x}(t),\;w(t)\}\in R^{7n+n_{w}}$ and exploit $\Gamma S_{2}\Gamma \leq {\bar{\tau }}^{2}S_{2}$. In line with Lemma 2.1 and Lemma 2.2, the stability conditions for the CLS (11) are given by the LMIs (19), (21), (35), and


$$\begin{array}{c} \begin{array}{cc} & 0>\left[\begin{array}{cccc} {\tilde{\Phi }}_{\kappa }(t)+h_{k}{\tilde{\Phi }}_{t} & \left(*\right) & \left(*\right) & \left(*\right)\\ D^{T}(\theta )G\Xi_{0} & -\gamma^{2}I & 0 & 0\\ C(\theta )e_{1} & 0 & -I & 0\\ \bar{\tau }(N_{1}\Xi_{5}+N_{2}\Xi_{6}+N_{3}\Xi_{7}) & 0 & 0 & -U \end{array}\right],\;\forall \kappa \in N_{2^{m}}\;\text{and}\;t\in \{t_{k},t_{k+1}\} \end{array} \end{array}$$ (53)

where


$$\begin{array}{cc} & {\tilde{\Phi }}_{\kappa }(t)=e_{1}^{T}\sigma P(\theta )e_{1}+\text{He}\{e_{1}^{T}P(\theta )e_{7}+e_{1}^{T}\dot{P}(\theta )e_{1}+\Xi_{0}^{T}G^{T}(A(\theta )e_{1}+{\bar{B}}_{\kappa }(\theta ,\hat{\theta })e_{5}-e_{7})\}\\ & \;+e_{1}^{T}S_{1}e_{1}-e_{6}^{T}S_{1}e_{6}+e_{7}^{T}\tau^{2}S_{2}e_{7}-(e_{1}^{T}-e_{5}^{T})S_{2}(e_{1}-e_{5})\\ & \;-(e_{5}^{T}-e_{6}^{T})S_{2}(e_{5}-e_{6})+e_{5}^{T}Ue_{5}+e_{7}^{T}R_{6}e_{7}\\ & \;+\text{He}\{-\Xi_{1}^{T}(t)W_{1}\Xi_{2}+\Xi_{3}^{T}(t)W_{2}\Xi_{4}+e_{7}^{T}\tau R_{1}e_{1}+e_{5}^{T}\tau R_{2}e_{7}\}\\ & \;+\text{He}\{(e_{1}^{T}-e_{5}^{T})R_{4}e_{7}+e_{7}^{T}R_{4}e_{1}+e_{7}^{T}R_{5}e_{5}-(e_{5}^{T}-e_{6}^{T})M(e_{1}-e_{5})\}\\ & \;-\text{He}\{e_{2}^{T}N_{1}\Xi_{5}+e_{3}^{T}N_{2}\Xi_{6}+e_{4}^{T}N_{3}\Xi_{7}\}\\ & {\tilde{\Phi }}_{t_{k}}=\text{He}\{\Xi_{1d}^{T}W_{1}\Xi_{2}+\Xi_{1}^{T}(t_{k})W_{1}\Xi_{2d}+e_{5}^{T}N_{3}\Xi_{7}\}\\ & {\tilde{\Phi }}_{t_{k+1}}=\text{He}\{\Xi_{3d}^{T}W_{2}\Xi_{4}+\Xi_{3}^{T}(t_{k+1})W_{2}\Xi_{4d}-e_{5}^{T}N_{2}\Xi_{6}\} \end{array}$$

Let us define $\bar{G}=G^{-1}$, congruence matrices ${\bar{G}}_{i}=\text{diag}\underset{i\text{ elements}}{\underbrace{\left\{\bar{G},\cdots ,\bar{G}\right\}}}$, and the following replacement variables:


$$\begin{array}{cc} & \bar{P}(\theta )={\bar{G}}^{T}P(\theta )\bar{G},\;\dot{\bar{P}}(\theta )={\bar{G}}^{T}\dot{P}(\theta )\bar{G},\;{\bar{S}}_{1}={\bar{G}}^{T}S_{1}\bar{G},\;{\bar{S}}_{2}={\bar{G}}^{T}S_{2}\bar{G},\;\bar{M}={\bar{G}}^{T}M\bar{G}\\ & {\bar{W}}_{1}={\bar{G}}_{2}^{T}W_{1}{\bar{G}}_{3},\;{\bar{W}}_{2}={\bar{G}}_{2}^{T}W_{2}{\bar{G}}_{3},\;{\bar{R}}_{\ell }={\bar{G}}^{T}R_{\ell }\bar{G},\;{\bar{N}}_{1}={\bar{G}}^{T}N_{1}{\bar{G}}_{2}\\ & {\bar{N}}_{2}={\bar{G}}^{T}N_{2}{\bar{G}}_{2},\;{\bar{N}}_{3}={\bar{G}}^{T}N_{3}{\bar{G}}_{2},\;\bar{U}={\bar{G}}^{T}U\bar{G},\;\bar{F}(\hat{\theta })=F(\hat{\theta })\bar{G},\;\bar{K}(\hat{\theta })=K(\hat{\theta })\bar{G}. \end{array}$$

With the transformed variables defined as above, congruent pre- and post-multiplication of (19) by ${\bar{G}}_{3}$, (21) by ${\bar{G}}_{2}$, (35) by ${\bar{G}}_{2}$, and (53) by ${\bar{G}}_{8}$ results in (46), (47), (48), and (49), respectively. ■

The following theorem presents the relaxation technique for PLMI-based stabilization conditions in Lemma 2.3.

**Theorem 2.1.** For given positive scalars σ, γ, $\bar{w}$, $\bar{u}$, $\underset{―}{\tau }$, $\tau_{i}$, for $i\in N_{n}$, and $\alpha_{s}$, for $s\in N_{r}$, CLS (11) is asymptotically stable if there exist matrices $0<{\bar{P}}_{i}={\bar{P}}_{i}^{T}\in R^{n\times n}$, $0<{\bar{S}}_{1}={\bar{S}}_{1}^{T}\in R^{n\times n}$, $0<{\bar{S}}_{2}={\bar{S}}_{2}^{T}\in R^{n\times n}$, ${\bar{R}}_{\ell }={\bar{R}}_{\ell }^{T}\in R^{n\times n}$, for ℓ∈{1,2,⋯,6}, $\bar{M}={\bar{M}}^{T}\in R^{n\times n}$, ${\bar{W}}_{1}\in R^{2n\times 3n}$, and ${\bar{W}}_{2}\in R^{2n\times 3n}$, ${\bar{N}}_{1}\in R^{n\times 2n}$, ${\bar{N}}_{2}\in R^{n\times 2n}$, ${\bar{N}}_{3}\in R^{n\times 2n}$, $0<\bar{U}={\bar{U}}^{T}\in R^{n\times n}$, $0<\bar{G}\in R^{n\times n}$, ${\bar{F}}_{j}\in R^{m\times n}$, ${\bar{K}}_{j}\in R^{m\times n}$, $X_{s}=X_{s}^{T}\in R^{n\times n}$, and $Y_{is}=Y_{is}^{T}\in R^{n\times n}$, such that the following conditions are satisfied, for $\kappa \in N_{2^{m}}$, $\ell \in N_{m}$, $t\in \{t_{k},t_{k+1}\}$, $h_{k}\in \{\underset{―}{\tau },\bar{h}\}$, and $i\in N_{r}$: LMIs (46) and (47), and


$$\begin{array}{c} \begin{array}{cc} & 0\leq \left[\begin{array}{cc} {\bar{P}}_{i} & \left(*\right)\\ \Lambda_{\ell }{\bar{K}}_{i} & \nu_{\ell } \end{array}\right] \end{array} \end{array}$$ (54)

$$\begin{array}{c} \begin{array}{cc} & 0>{\bar{\Omega }}_{\kappa ii}(t) \end{array} \end{array}$$ (55)

$$\begin{array}{c} \begin{array}{cc} & 0>{\bar{\Omega }}_{\kappa ii}(t)+\sum_{j=1,j\text{≠}i}^{r}\frac{1}{2}({\bar{\Omega }}_{\kappa ij}(t)+{\bar{\Omega }}_{\kappa ji}(t)) \end{array} \end{array}$$ (56)

where


$$\begin{array}{cc} & {\bar{\Omega }}_{\kappa ij}(t)=\left[\begin{array}{cccccc} {\bar{\Phi }}_{\kappa ij}+{\bar{\Phi }}_{0}(t)+h_{k}{\bar{\Phi }}_{t}+X_{i}\;\;\;\;\; & \left(*\right) & \left(*\right) & \left(*\right) & \left(*\right) & \left(*\right)\\ D^{T}(\theta )\Xi_{0} & \;\;-\gamma^{2}I & 0 & 0 & 0 & 0\\ C(\theta )\bar{G}e_{1} & 0 & -I & 0 & 0 & 0\\ \bar{\tau }({\bar{N}}_{1}\Xi_{5}+{\bar{N}}_{2}\Xi_{6}+{\bar{N}}_{3}\Xi_{7}) & 0 & 0 & -\bar{U}\; & 0 & 0\\ {\left[({\bar{P}}_{s}-{\bar{P}}_{r})e_{1}\right]}_{s\in N_{r-1}} & 0 & 0 & \;0\; & \;\;\;\;\;\;\;-{\left[X_{s}\right]}_{s\in N_{r-1}}^{d}\;\;\;\;\; & 0\\ \;\;\;{\left[B_{i}(\Xi_{\kappa }\Delta {\bar{F}}_{s}+{\bar{\Xi }}_{\kappa }\Delta {\bar{K}}_{s})e_{5}\right]}_{s\in N_{r-1}}\;\;\;\;\; & 0 & 0 & \;0\; & 0 & \;\;\;\;\;-{\left[Y_{is}\right]}_{s\in N_{r-1}}^{d}\;\;\; \end{array}\right] \end{array}$$

in which ${\bar{\Phi }}_{0}(t)$ and ${\bar{\Phi }}_{t}$ are defined in Lemma 2.3, and


$$\begin{array}{cc} & {\bar{\Phi }}_{\kappa ij}=e_{1}^{T}\sigma {\bar{P}}_{i}e_{1}+\text{He}\{e_{1}^{T}{\bar{P}}_{i}e_{7}+\Xi_{0}^{T}(A_{i}\bar{G}e_{1}+B_{i}(\Xi_{\kappa }{\bar{F}}_{j}+{\bar{\Xi }}_{\kappa }{\bar{K}}_{j})e_{5})\}\\ & X_{i}=\sum_{s=1}^{r-1}(\frac{\alpha_{s}^{2}}{4}e_{1}^{T}X_{s}e_{1}+\beta_{s}^{2}\Xi_{0}^{T}Y_{is}\Xi_{0}),\;\Delta {\bar{F}}_{s}={\bar{F}}_{s}-{\bar{F}}_{r},\;\Delta {\bar{K}}_{s}={\bar{K}}_{s}-{\bar{K}}_{r}. \end{array}$$

Moreover, the control gains are reconstructed by $F_{i}={\bar{F}}_{i}{\bar{G}}^{-1}$.

*Proof:* By letting


$$\begin{array}{c} \bar{P}(\theta )=\sum_{i=1}^{r}\theta_{i}{\bar{P}}_{i},\;\dot{\bar{P}}(\theta )=\sum_{i=1}^{r}{\dot{\theta }}_{i}{\bar{P}}_{i},\;\bar{F}(\theta )=\sum_{i=1}^{r}\theta_{i}{\bar{F}}_{i},\;\bar{K}(\theta )=\sum_{i=1}^{r}\theta_{i}{\bar{K}}_{i} \end{array}$$ (57)

it is given from (13) that


$$\begin{array}{c} \begin{array}{cc} & \dot{\bar{P}}(\theta )=\sum_{s=1}^{r-1}{\dot{\theta }}_{s}{\bar{P}}_{s}+{\dot{\theta }}_{r}{\bar{P}}_{r}=\sum_{s=1}^{r-1}{\dot{\theta }}_{s}({\bar{P}}_{s}-{\bar{P}}_{r}) \end{array} \end{array}$$ (58)

$$\begin{array}{c} \begin{array}{cc} & \bar{F}(\bar{\theta })=\sum_{s=1}^{r-1}{\bar{\theta }}_{s}{\bar{F}}_{s}+{\bar{\theta }}_{r}{\bar{F}}_{r}=\sum_{s=1}^{r-1}{\bar{\theta }}_{s}\Delta {\bar{F}}_{s} \end{array} \end{array}$$ (59)

$$\begin{array}{c} \begin{array}{cc} & \bar{K}(\bar{\theta })=\sum_{s=1}^{r-1}{\bar{\theta }}_{s}{\bar{K}}_{s}+{\bar{\theta }}_{r}{\bar{K}}_{r}=\sum_{s=1}^{r-1}{\bar{\theta }}_{s}\Delta {\bar{K}}_{s}. \end{array} \end{array}$$ (60)

From (59)–(60), condition (49) is rearranged as follows, for $\kappa \in N_{2^{m}}$, $t\in \{t_{k},t_{k+1}\}$, and $h_{k}\in \{\underset{―}{\tau },\bar{h}\}:$


$$\begin{array}{c} 0>\Omega_{\kappa }(\theta ,t)\;+\;\Upsilon^{T}(\sum_{s=1}^{r-1}{\dot{\theta }}_{s}e_{1}^{T}({\bar{P}}_{s}-{\bar{P}}_{r})e_{1}\;+\;\sum_{s=1}^{r-1}{\bar{\theta }}_{s}He\{\Xi_{10}^{T}B(\theta )(\Xi_{\kappa }\Delta {\bar{F}}_{s}\;+\;{\bar{\Xi }}_{\kappa }\Delta {\bar{K}}_{s})e_{5}\})\Upsilon \end{array}$$ (61)

where


$$\begin{array}{cc} & \Omega_{\kappa }(\theta ,t)=\left[\begin{array}{cccc} {\bar{\Phi }}_{\kappa }(\theta )+{\bar{\Phi }}_{0}(t)+h_{k}{\bar{\Phi }}_{t} & \left(*\right) & \left(*\right) & \left(*\right)\\ D^{T}(\theta )\Xi_{0} & -\gamma^{2}I & 0 & 0\\ C(\theta )\bar{G}e_{1} & 0 & -I & 0\\ \bar{\tau }({\bar{N}}_{1}\Xi_{5}+{\bar{N}}_{2}\Xi_{6}+{\bar{N}}_{3}\Xi_{7}) & 0 & 0 & -\bar{U} \end{array}\right],\;\Upsilon =\left[\begin{array}{cc} I_{8n} & 0_{8n\times n} \end{array}\right]. \end{array}$$

Since (56) implies $X_{s}>0$ and $Y_{s}(\theta )=\sum_{i=1}^{r}\theta_{i}Y_{is}>0$, condition (14) implies


$$\begin{array}{c} \begin{array}{cc} & {\dot{\theta }}_{s}e_{1}^{T}({\bar{P}}_{s}-{\bar{P}}_{r})e_{1}\leq \frac{\alpha_{s}^{2}}{4}e_{1}^{T}X_{s}e_{1}+e_{1}^{T}({\bar{P}}_{s}^{T}-{\bar{P}}_{r}^{T})X_{s}^{-1}({\bar{P}}_{s}-{\bar{P}}_{r})e_{1} \end{array} \end{array}$$ (62)

$$\begin{array}{c} \begin{array}{cc} & {\bar{\theta }}_{s}He\{\Xi_{10}^{T}B(\theta )F_{s}e_{5}\}\leq \beta_{s}^{2}\Xi_{10}^{T}Y_{s}(\theta )\Xi_{10}+e_{5}^{T}F_{s}^{T}B^{T}(\theta )Y_{s}^{-1}(\theta )B(\theta )F_{s}e_{5} \end{array} \end{array}$$ (63)

where $F_{s}=\Xi_{\kappa }\Delta {\bar{F}}_{s}+{\bar{\Xi }}_{\kappa }\Delta {\bar{K}}_{s}$. Accordingly, condition (61) is ensured by


$$\begin{array}{cc} 0 & >\Omega_{g}(\theta )+\Upsilon^{T}\sum_{s=1}^{r-1}(\frac{\alpha_{s}^{2}}{4}e_{1}^{T}X_{s}e_{1}+\beta_{s}^{2}\Xi_{10}^{T}Y_{s}(\theta )\Xi_{10})\Upsilon \\ & \;+\Upsilon^{T}(\sum_{s=1}^{r-1}e_{1}^{T}({\bar{P}}_{s}^{T}-{\bar{P}}_{r}^{T})X_{s}^{-1}({\bar{P}}_{s}-{\bar{P}}_{r})e_{1})\Upsilon \\ & \;+\Upsilon^{T}(\sum_{s=1}^{r-1}e_{5}^{T}F_{s}^{T}B^{T}(\theta )Y_{s}^{-1}(\theta )B(\theta )F_{s}e_{5})\Upsilon \end{array}$$

which is transformed by the Schur complement into


$$\begin{array}{c} \begin{array}{cc} & 0>\left[\begin{array}{ccc} \Omega_{\kappa }(\theta ,t)+\Upsilon^{T}X(\theta )\Upsilon & \left(*\right) & \left(*\right)\\ {\left[({\bar{P}}_{s}-{\bar{P}}_{r})e_{1}\Upsilon \right]}_{s\in N_{r-1}} & -{\left[X_{s}\right]}_{s\in N_{r-1}}^{d} & \left(*\right)\\ {\left[B(\theta )F_{s}e_{5}\Upsilon \right]}_{s\in N_{r-1}} & 0 & -{\left[Y_{s}(\theta )\right]}_{s\in N_{r-1}}^{d} \end{array}\right]\\ & =\sum_{i=1}^{r}\sum_{j=1}^{r}\theta_{i}\theta_{j}{\bar{\Omega }}_{gij} \end{array} \end{array}$$ (64)

where $X(\theta )=\sum_{i=1}^{r}\theta_{i}X_{i}$. As a result, with the aid of Lemma 1.3, the relaxed conditions of (64) are given as (56).

## 4 Illustrative examples

This section provides two numerical examples: the first computes the MASI for multi-rate SDCSs using Lemma 2.1, and the second designs the controller gain for a truck-trailer system with a specified multi-rate sampling interval using Theorem 2.1. The LMI conditions in Lemma 2.1 and Theorem 2.1 are solved using the LMI solver in the Robust Control Toolbox of MATLAB R2024a, running on a desktop PC with an Intel Core i5-10400F CPU and 16 GB RAM under Windows 11 Education.

**Example 1.** Consider the multi-rate SDCS with *n* = 2 (refer to [19]):


$$\begin{array}{c} \begin{array}{cc} & A=\left[\begin{array}{cc} 0 & 1\\ 0 & -0.1 \end{array}\right],\;B=\left[\begin{array}{cc} 0 & 0.1 \end{array}\right]\\ & F=\left[\begin{array}{cc} -3.17 & -11.5 \end{array}\right]. \end{array} \end{array}$$ (65)

**Fig 2:**
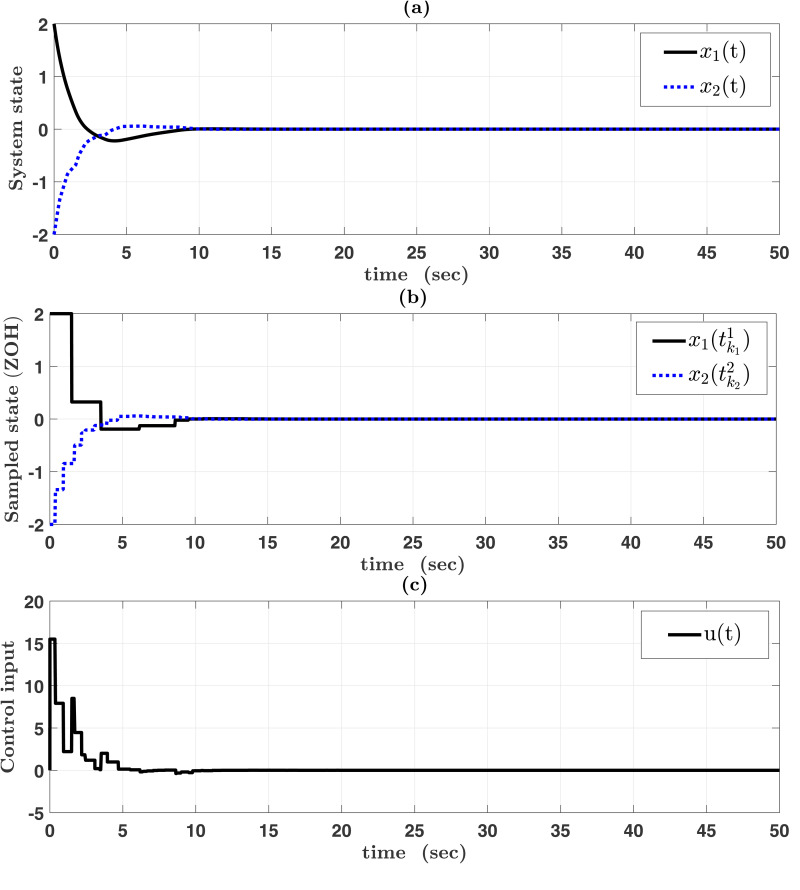
Simulation result for system (65) with $\tau_{1}=3$ and $\tau_{2}=0.90$.

The parameters in Lemma 2.1 are given as $\sigma =10^{-6}$ and $\underset{―}{\tau }=10^{-3}$, and the sampling interval of the first state is chosen as $\tau_{1}\in \{2,3\}$. For a fair comparison, the same system matrices *A*, *B* and controller gain *F* in (65) are used for the proposed Lemma 2.1 and all compared methods [19–21]; hence, the reported MASI $\tau_{2}$ reflects only the conservatism of each stability condition rather than any difference in controller design. For comparison, Tables 1 and 2 shows the MASI of the second state $\tau_{2}$ obtained by 19, Theorem 1], [20, Theorem 1], [21, Theorem 1], and Lemma 2.1. Accordingly, Lemma 2.1 provides larger MASIs than the competing methods [19–21]. Specifically, compared with the most recent result in [21, Theorem 1], Lemma 2.1 increases the MASI $\tau_{2}$ by approximately 26.6% for $\tau_{1}=2$ and by about 4.6% for $\tau_{1}=3$. It should be noted that when the sampling interval $\tau_{1}$ increases, the MASI $\tau_{2}$ generally decreases due to the stability constraints of the multi-rate sampled-data system. This phenomenon has also been observed in the literature [19, Theorem 1] and [20, Theorem 1]. In addition, Lemma 2.1 achieves significantly lower computational complexity than the existing methods. As shown in Tables 1 and 2, the proposed method consistently yields shorter computational times in both cases. Specifically, for $\tau_{1}=2$, the proposed method requires 0.3169 s, which is approximately 9.4 times faster than [21, Theorem 1] (2.9670 s). For $\tau_{1}=3$, the computational time is reduced from 2.9701 s to 0.2581 s, corresponding to a speedup of approximately 11.5 times. To evaluate the trade-off introduced by the slack matrices *N*_1_, *N*_2_, and *N*_3_, we compare the full formulation of Lemma 2.1 with the reduced case $\left(N_{i}\equiv 0\right)$. As shown in Tables 1 and 2, the slack matrices improve the MASI by approximately 12.1% and 5.6% for $\tau_{1}=2$ and $\tau_{1}=3$, respectively, at the cost of increasing the computational time by a factor of roughly 2.6−2.8. Since the number of slack variables grows with the system state dimension, the two formulations provide a flexible trade-off between conservatism and computational cost. The full formulation is preferable for low-order systems, where the additional decision variables incur negligible overhead while minimizing conservatism. In contrast, the reduced formulation $\left(N_{i}\equiv 0\right)$ is more suitable for higher-order systems, as it substantially limits the growth of decision variables at the expense of a slightly smaller MASI. Next, for $\tau_{1}=3$, $\tau_{2}=0.90$, and $x(t_{0})=\text{col}\{2,-2\}$, the trajectories of *x*(*t*), $\bar{x}(t_{k})$, and *u*(*t*) are shown in Fig 2-(a), (b), and (c), respectively. As shown in Fig 2, the system state converges to the equilibrium point, confirming the validity of the MASI reported in Table 1.

**Table 1:** Comparison of MASI $\tau_{2}$ for $\tau_{1}=2$.

methods	MASI $\tau_{2}$	Computational time (s)
[19, Theorem 1]	0.57	0.0910
[20, Theorem 1]	0.69	6.2755
[21, Theorem 1]	0.8691	2.9670
Lemma 2.1	1.1003	0.3169
Lemma 2.1 $\left(N_{i}\equiv 0\right)$	0.9812	0.1139

**Table 2:** Comparison of MASI $\tau_{2}$ for $\tau_{1}=3$.

methods	MASI $\tau_{2}$	Computational time (s)
[19, Theorem 1]	0.17	0.0839
[20, Theorem 1]	0.48	5.7408
[21, Theorem 1]	0.8691	2.9701
Lemma 2.1	0.9090	0.2581
Lemma 2.1 $\left(N_{i}\equiv 0\right)$	0.8605	0.0979

**Fig 3:**
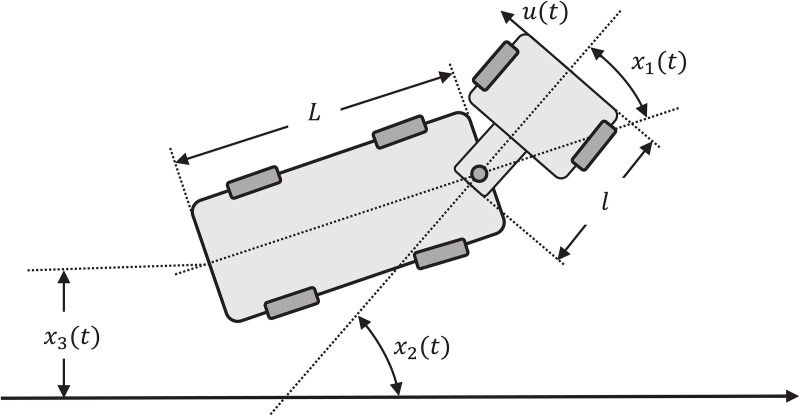
Schematic diagram of truck-trailer system.

**Example 2.** Let us consider a truck-trailer system shown in Fig 3 (refer to [40]), where *x*_1_(*t*), *x*_2_(*t*), and *x*_3_(*t*) denote the angular between trailer and truck (*rad*), the angle of trailer (*rad*), and the vertical position of the trailer (*m*), respectively; and *u*(*t*) denotes the steering angle of the front wheels, which serves as the control input. Specifically, the bound $|x_{1}(t)|\leq \pi /2$ defines the region within which the backing maneuver remains controllable. Although articulation angles beyond π/2 are physically possible, the steering authority of the front wheels becomes insufficient to recover the trailer to the desired path once the relative angle exceeds this threshold. Meanwhile, a heading error exceeding π/2 points the trailer away from the target path, rendering the backing maneuver ill-posed; $|x_{2}(t)|\leq \pi /2$ thus confines the analysis to the envelope in which convergence to the desired path is achievable. Furthermore, the steering input *u*(*t*) is bounded by the actuator saturation |u(t)|≤π/2. As in [40], the dynamics model of the truck-trailer system is given as follows:


$$\begin{array}{c} \begin{array}{cc} {\dot{x}}_{1}(t) & =-\frac{\upsilon \bar{\tau }}{L\delta_{0}}x_{1}(t)+\frac{\upsilon \bar{\tau }}{l\delta_{0}}u(t)+0.5w(t) \end{array} \end{array}$$ (66)

$$\begin{array}{c} \begin{array}{cc} {\dot{x}}_{2}(t) & =\frac{\upsilon \bar{\tau }}{L\delta_{0}}x_{1}(t)+0.5w(t) \end{array} \end{array}$$ (67)

$$\begin{array}{c} \begin{array}{cc} {\dot{x}}_{3}(t) & =\frac{\upsilon \bar{\tau }}{\delta_{0}}\sin(x_{2}(t)+\frac{\upsilon \bar{\tau }}{2L}x_{1}(t)) \end{array} \end{array}$$ (68)

where the model parameters are *L* = 5.5 (*m*), *l* = 2.8 (*m*), υ=−1.0(m/s), $\bar{\tau }=2.0(s)$, $\delta_{0}=0.5(s)$, and *w*(*t*) denotes the external disturbances satisfying the energy bound $\bar{w}=1$. Then, by letting $\xi (t)=x_{2}(t)+\frac{\upsilon \bar{\tau }}{2L}x_{1}(t)$, $\mu =10^{-2}/\pi$, and defining the FBFs as follows:


$$\begin{array}{cc} & \theta_{1}(t)=\left\{ \begin{array}{cc} \frac{\sin(\xi (t))-\mu \xi (t)}{\xi (t)(1-\mu )}, & \;\text{if}\;\xi (t)\text{≠}0\\ 1,\; & \;\text{if}\;\xi (t)=0 \end{array}\right.\\ & \theta_{2}(t)=1-\theta_{1}(t) \end{array}$$

The truck–trailer system with performance output *z*(*t*) = *x*_3_(*t*) is represented in the form of (1) with the following system matrices:


$$\begin{array}{cc} & A_{1}=\left[\begin{array}{ccc} -\frac{\upsilon \bar{\tau }}{L\delta_{0}}\; & \;0\; & \;0\\ \frac{\upsilon \bar{\tau }}{L\delta_{0}}\; & \;0\; & \;0\\ \frac{\upsilon^{2}{\bar{\tau }}^{2}}{2L\delta_{0}} & \frac{\upsilon \bar{\tau }}{\delta_{0}}\; & \;0 \end{array}\right]\;,\;A_{2}=\left[\begin{array}{ccc} -\frac{\upsilon \bar{\tau }}{L\delta_{0}}\; & \;0\; & \;0\\ \frac{\upsilon \bar{\tau }}{L\delta_{0}}\; & \;0\; & \;0\\ \frac{\mu \upsilon^{2}{\bar{\tau }}^{2}}{2L\delta_{0}} & \frac{\mu \upsilon \bar{\tau }}{\delta_{0}}\; & \;0 \end{array}\right]\;,\;B_{i}=\left[\begin{array}{c} \frac{\upsilon \bar{\tau }}{l\delta_{0}}\\ 0\\ 0 \end{array}\right]\\ & D_{i}^{T}=\left[\begin{array}{ccc} 0.5 & 0 & 0\\ 0 & 0.5 & 0 \end{array}\right],\;C_{i}=\left[\begin{array}{ccc} 0 & 0 & 1 \end{array}\right]. \end{array}$$

The time derivative of $\theta_{1}(t)$ is given as follows:


$$\begin{array}{c} {\dot{\theta }}_{1}(t)=G(\xi )\;\dot{\xi }(t),\;G(\xi )≜\frac{d\theta_{1}}{d\xi }=\frac{1}{1-\mu }\;\frac{\xi (t)\cos(\xi (t))-\sin(\xi (t))}{\xi^{2}(t)}. \end{array}$$ (69)

From the operating region $x_{1}(t),x_{2}(t)\in [-\pi /2,\pi /2]$, the premise variable is bounded by


$$\begin{array}{c} |\xi (t)|\leq |x_{2}(t)|+\frac{|v|\bar{\tau }}{2L}\;|x_{1}(t)|\leq 1.856. \end{array}$$ (70)

Since $h(\xi )=(\xi (t)\cos(\xi (t))-\sin(\xi (t)))/(\xi^{2}(t))$ is odd and |h(ξ)| is monotonically increasing on (0,1.856], it is given that


$$\begin{array}{c} |G(\xi )|\leq \frac{\left|h(\xi_{\max})\right|}{1-\mu }\leq 0.432. \end{array}$$ (71)

Meanwhile, differentiating ξ and substituting the dynamics (66) and (67) yields


$$\begin{array}{c} \dot{\xi }(t)={\dot{x}}_{2}(t)+\frac{v\bar{\tau }}{2L}\;{\dot{x}}_{1}(t)=\frac{v\bar{\tau }}{L\delta_{0}}\;\left(1-\frac{v\bar{\tau }}{2L}\right)x_{1}(t)+\frac{v^{2}{\bar{\tau }}^{2}}{2Ll\delta_{0}}\;u(t)+0.1\;\left(1+\frac{v\bar{\tau }}{2L}\right)w(t). \end{array}$$ (72)

Bounding (72) over $x_{1}(t),u(t)\in [-\pi /2,\pi /2]$ and |w(t)|≤1 gives $|\dot{\xi }(t)|\leq 1.878$. Accordingly, the required derivative bound is given as follows:


$$\begin{array}{c} |{\dot{\theta }}_{1}(t)|=|{\dot{\theta }}_{2}(t)|\leq |G(\xi )|\;|\dot{\xi }(t)|\leq 0.432\times 1.878\approx 0.811. \end{array}$$ (73)

Physically, the articulation angle *x*_1_(*t*) changes rapidly because it is directly affected by the steering input *u*(*t*) and the motion of the truck; therefore, it requires a higher sampling rate. In contrast, the variable *x*_2_(*t*) evolves more slowly since it depends on the overall vehicle motion and accumulates the effect of *x*_1_(*t*) over time. As a result, *x*_2_(*t*) can be sampled at a lower rate. For setting $\sigma =10^{-3}$, $\underset{―}{\tau }=10^{-3}$, $\lambda_{1}=0.5$, $\lambda_{2}=0.5$, $\tau_{1}=0.1$, $\tau_{3}=0.1$, $\gamma^{2}=0.5$ and $\alpha_{s}=0.811$, for all $s\in N_{r}$, Theorem 2.1 yields the MASI $\tau_{2}=0.3$, and the following control gain:


$$\begin{array}{cc} & F_{1}=\left[\begin{array}{ccc} 1.7753 & -0.9103 & 0.0832 \end{array}\right]\\ & F_{2}=\left[\begin{array}{ccc} 1.7561 & -0.8684 & 0.0794 \end{array}\right]. \end{array}$$

**Fig 4:**
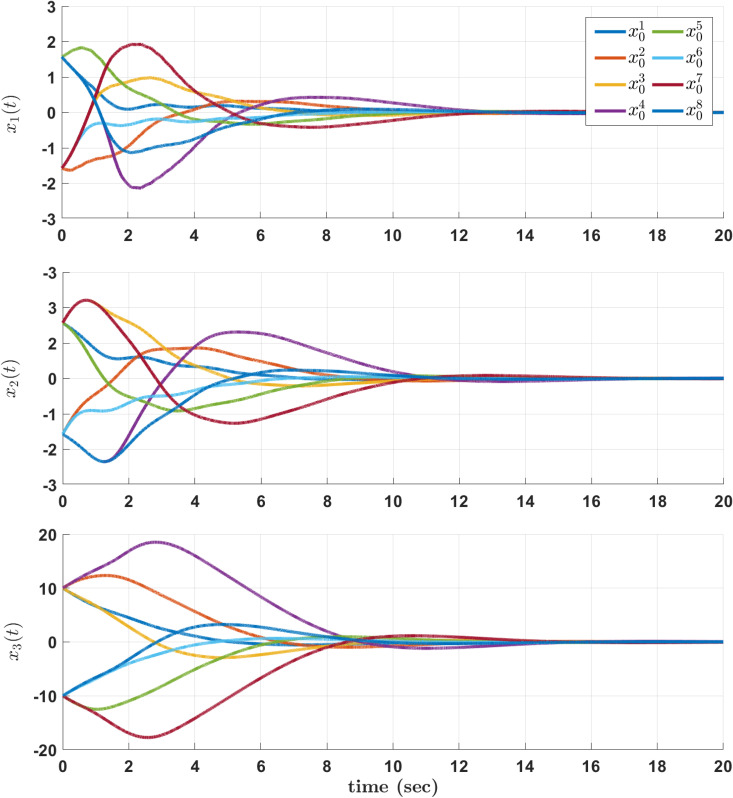
Closed-loop state responses for different initial conditions $x_{1}(t_{0}),\;x_{2}(t_{0})\in \{-\pi /2,\;\pi /2\}$ and $x_{3}(t_{0})\in \{-10,\;10\}$.

**Fig 5:**
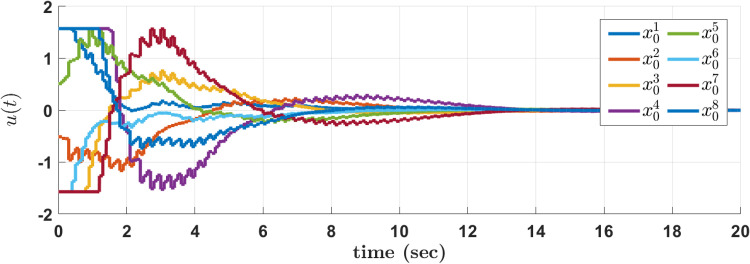
Sampled-data control input for different initial conditions $x_{1}(t_{0}),\;x_{2}(t_{0})\in \{-\pi /2,\;\pi /2\}$ and $x_{3}(t_{0})\in \{-10,\;10\}$.

**Fig 6:**
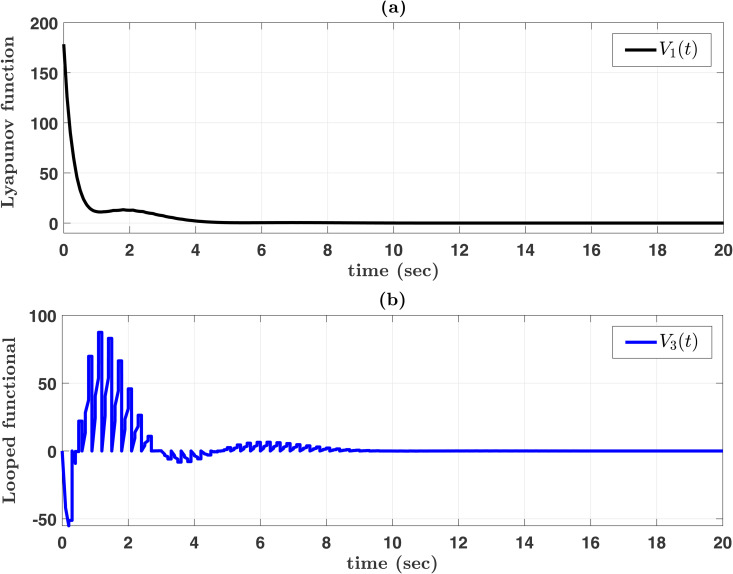
LKF components: (a) quadratic term *V*_1_(*t*) and (b) looped functional *V*_3_(*t*).

**Fig 7:**
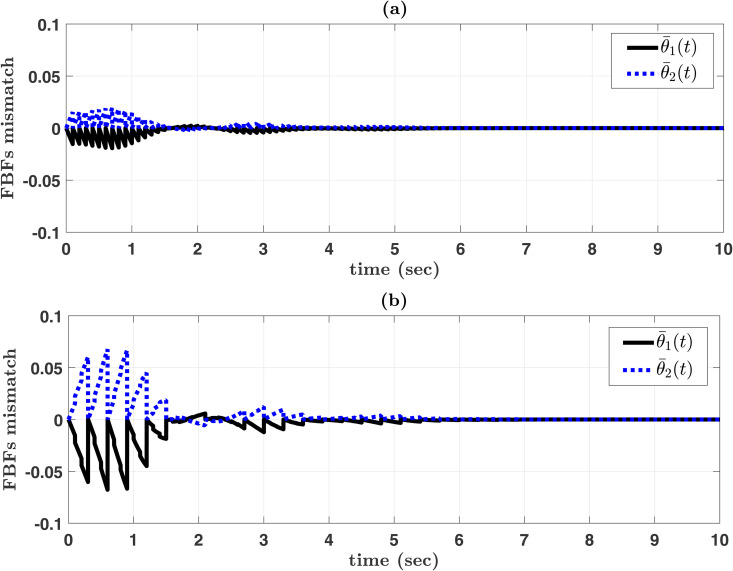
Mismatched FBFs for (a) single-rate sampling and (b) multi-rate sampling cases.

**Fig 8:**
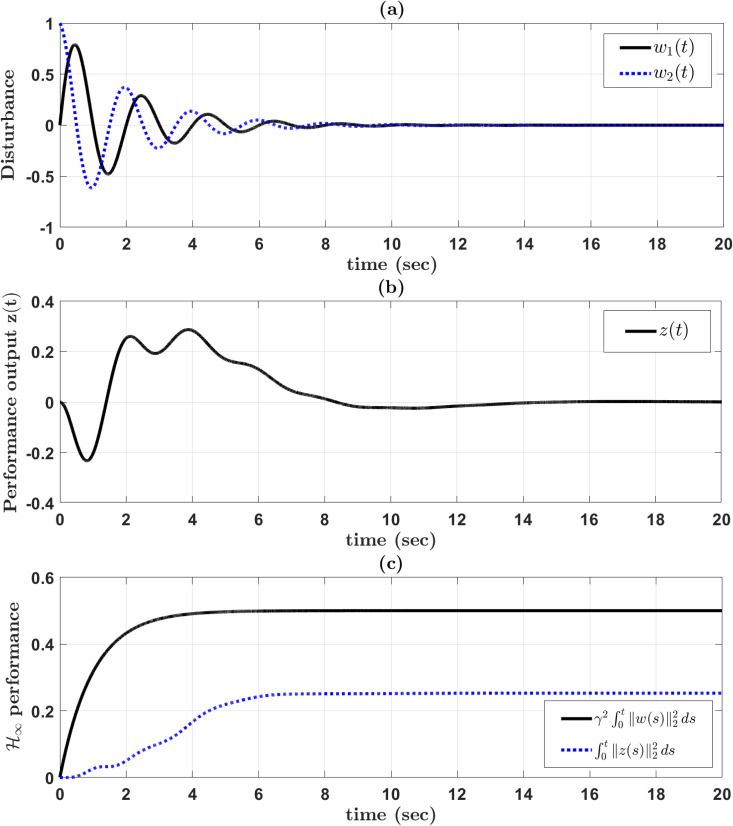
${ℋ}_{\infty }$ performance verification.

Based on the obtained controller gains, Fig 4 shows the closed-loop state responses from eight initial conditions at the corners of the operating region, i.e., $x_{1}(t_{0}),\;x_{2}(t_{0})\in \{-\pi /2,\;\pi /2\}$ and $x_{3}(t_{0})\in \{-10,\;10\}$ and external disturbance $w(t)=e^{-0.5t}[\sin(\pi t)\;\cos(\pi t){]}^{T}$. Despite the large initial conditions, all eight trajectories of *x*_1_(*t*), *x*_2_(*t*), and *x*_3_(*t*) converge asymptotically to the origin, demonstrating that the proposed con*t*roller s*t*abilizes *t*he system over the entire operating region. Fig 5 plots the sampled-data control input *u*(*t*) for eight initial conditions. During the initial transient, *u*(*t*) reaches the actuator sa*t*uration bound $\bar{u}=\pi /2$, consistent with the large initial sta*t*es at the boundary of the operating region. All trajectories satisfy |u(t)|≤π/2 for all t≥0, confirming that the admissible input constraint is respected under worst-case initialization. In addition, Fig 6 illustrates the time responses of the LKF components. As shown, the quadratic term *V*_1_(*t*) remains positive and decreases to zero. The looped functional *V*_3_(*t*) is not restricted to be positive definite, which introduces relaxation into the stability conditions. Nevertheless, *V*_3_(*t*) vanishes at each sampling instant, ensuring tha*t* the total LKF energy decreases mono*t*onically over each sampling interval and the overall stability is preserved. Furthermore, Fig 7 shows the time responses of the FBFs mismatch under (a) single-rate sampling with $\tau_{1}=\tau_{2}=0.1$ s and (b) multi-rate sampling with $\tau_{1}=0.1$ s and $\tau_{2}=0.3$ s. As expec*t*ed, the mismatch is more pronounced in the multi-rate case due to the heterogeneous sampling intervals across the membership function components, highlighting the necessity of explicitly addressing the FBFs mismatch in the stability analysis of multi-rate sampled-data T-S fuzzy systems. Finally, Fig 8 verifies the ${ℋ}_{\infty }$ performance of the closed-loop system under zero initial conditions, i.e., *x*(*t*_0_) = 0. As shown, the running integral of the regulated performance output energy remains strictly below the disturbances energy bound for all t≥0, confirming that the prescribed attenuation condition


$$\begin{array}{c} \int_{0}^{t}\|z(s){\|}_{2}^{2}\;ds\leq \gamma^{2}\int_{0}^{t}\|w(s){\|}_{2}^{2}\;ds \end{array}$$

is satisfied with $\gamma^{2}=0.5$.

## 5 Conclusions

This paper investigated less conservative stabilization criterion for MRSD T-S fuzzy systems by (i) reconstructing the looped-functional with delay-dependent terms, (ii) embedding the time-dependent discontinuous term in the LKF to exploit state-sampling patterns, and (iii) introducing the zero-equality augmentations with slack matrices. Then, we proposed an effective relaxation method that explicitly exploits mismatched FBFs inherent properties in the derivation of stabilization conditions formulated as LMIs. Two theoretical limitations warrant further investigation. First, the proposed framework requires an explicit bound $\alpha_{i}$ on the FBFs time derivative to be derived from the system dynamics prior to controller design, which is not always straightforward. A promising direction is to treat ${\dot{\theta }}_{i}(t)$ as a design parameter determined jointly with the controller gains within a local stabilization framework for T-S fuzzy systems, thereby unifying the bound estimation and the synthesis into a single co-design procedure. Second, the current LKF considers only sampling-induced delays, excluding transmission delays inherent in networked control systems. Incorporating both delay sources into a unified looped functional is a nontrivial extension, since the two delays have different structures and must be decoupled while preserving the multi-rate sampling pattern. Two examples are given to demonstrate that the proposed method yields larger MASI while reducing computational complexity. In future work, these limitations will be addressed by developing a local stabilization framework that jointly determines the FBFs derivative bound and the controller gains, and by extending the looped functional to incorporate both sampling-induced and transmission delays. The proposed framework will also be applied to UAV systems with a decoupled multi-rate control structure, where the attitude and position controllers operate at different sampling rates consistent with their respective physical timescales.
